# Supplementary material for: DNA minicircles as novel STAT3 decoy oligodeoxynucleotides endowed with anticancer activity in triple-negative breast cancer
Source: Mol Ther Nucleic Acids. 2022 Jun 22;29:162–75. doi: 10.1016/j.omtn.2022.06.012 (PMC9263874; doi:10.1016/j.omtn.2022.06.012)
Supplement: Document S1. Figures S1–S11 [file mmc1.pdf]

OMTN, Volume *29*

Supplemental information

DNA minicircles as novel STAT3 decoy oligodeoxynucleotides endowed with anticancer activity in triple-negative breast cancer

Geoffrey Casas, Federico Perche, Patrick Midoux, Chantal Pichon, and Jean-Marc Malinge

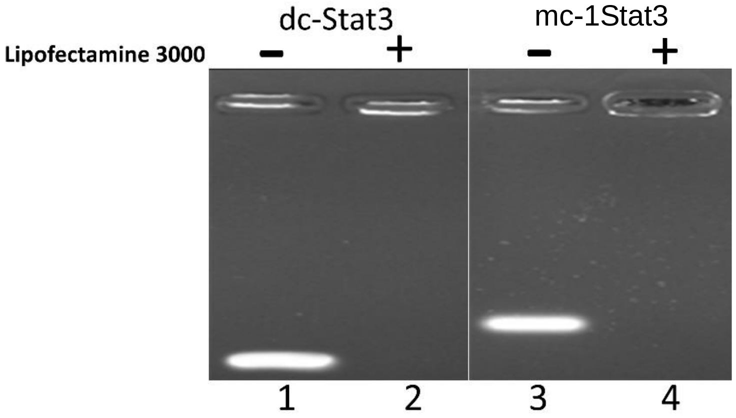

Figure S1. Gel retardation electrophoresis of linear (dc-Stat3) and MC (mc-1Stat3) DNA decoy confirms the condensation of these nucleic acids species with lipofectamine 3000. In the presence of this cationic liposome (lanes 2, 4), no more detectable band of free nucleic acid was observed while unformulated nucleic acids migrated freely as shown in lanes 1 and 3.

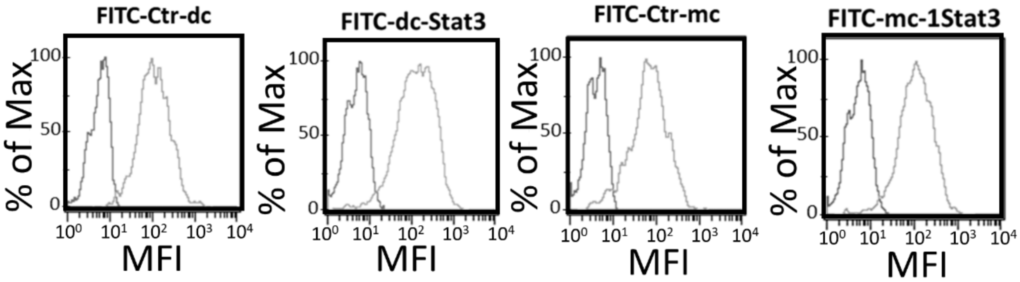

Figure S2. Cellular uptake is the same for formulated linear and DNA ODN. TNBC cancer cells were transfected in the presence of 0 nM (dark line) and 15 nM (light line) of various fluorescein-labeled linear and MCs ODNs as indicated on the panels. Flow cytometric analysis of cellular uptake were performed 6 hours after beginning treatment with lipofectamine 3000 formulated ODNs. These results show that the different specific ODNs used herein were equally taken up by the cells as their respective control ODN counterparts.

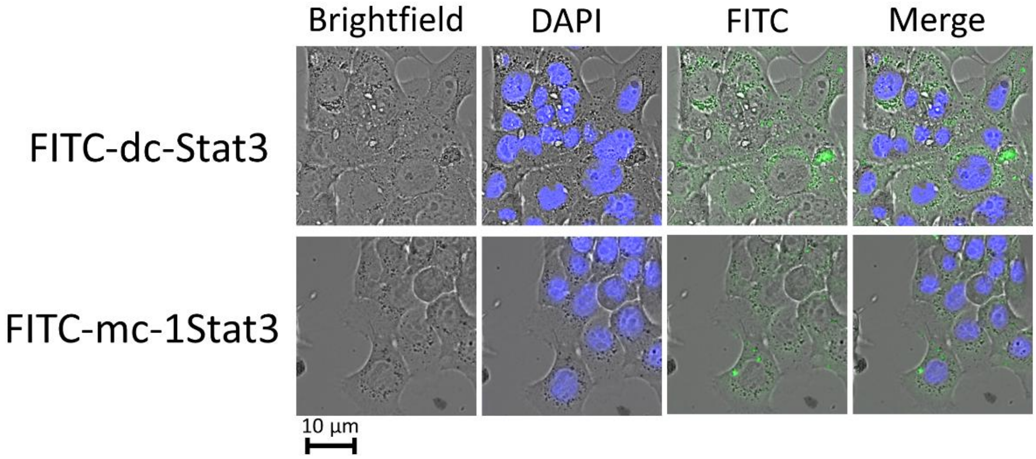

Figure S3. Comparative uptake of fluorescein-labeled dc-Stat3 and mc-1Stat3 by 4T1. 6 hours post-transfection with lipofectamine 3000 formulated ODN complexes (15 nM), confocal imaging was used with or without DAPI staining (blue) to compare intracellular delivery of both FITC-labeled linear decoy dc-Stat3 and minicircle mc-1Stat3 (green).

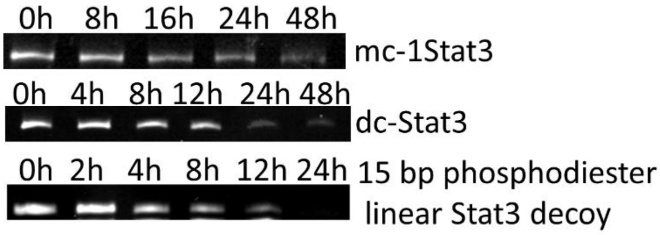

Figure S4. Biostability of 95 bp MC versus 15 bp double-stranded linear ODN in cell extract: image of denaturating gel electrophoresis showing time dependent stability of mc-1Stat3, dc-Stat3 and 15 bp phosphodiester linear STAT3 decoy in cellular extract. After incubation in cellular extract at 37°C, aliquots were taken as a function of time and loaded on denaturating polyacrylamide gel. Next, the bands were quantified following gel staining.

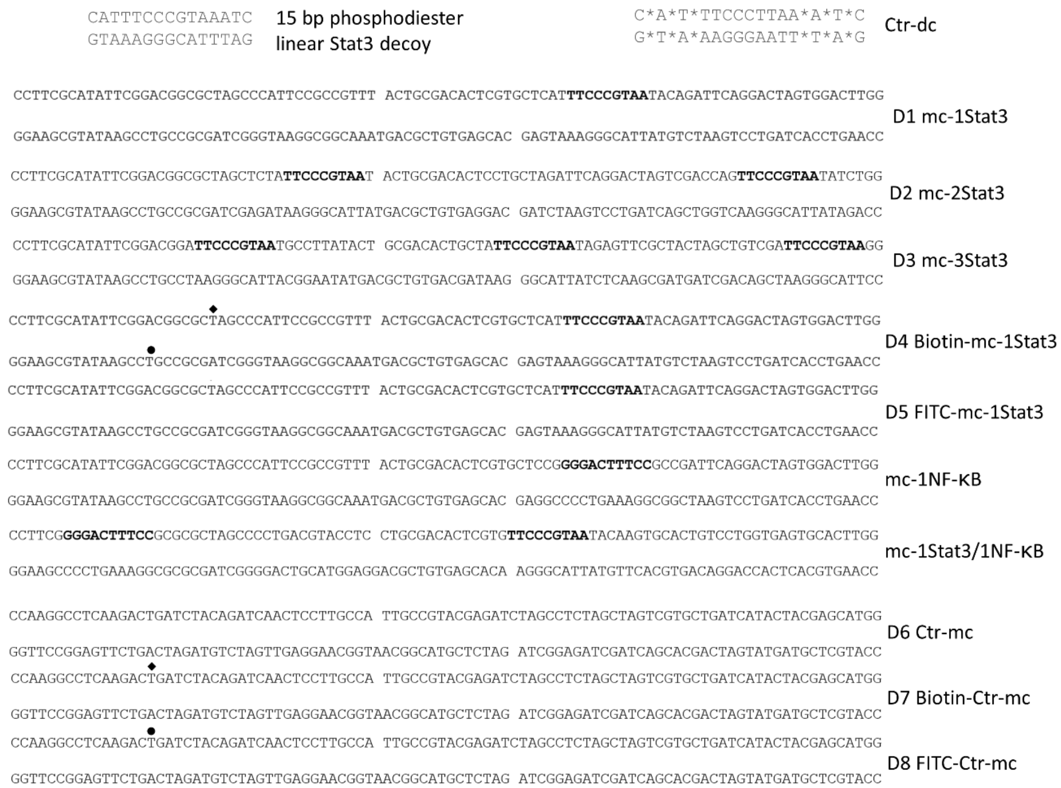

Figure S5. Sequence of DNA duplexes used in this study. 15 bp phosphodiester linear STAT3 decoy and Ctr-dc ODNs were used without further modification. Asteriks indicate phosphorothioate linkage present in linear Ctr-dc. Blunt-ended overlapping nicked 95 bp DNA duplexes oligonucleotide (D1 to D8) have been used for both the preparation of various minicircles (MCs) and two linear 95 pb DNA duplexes according to separate experimental procedure (see Material and Methods). D1 : mc1-Stat3 or 95 bp linear Stat3 DNA decoy; D2: mc-2Stat3; D3 :mc-3Stat3; D4: biotinylated mc-1Stat3; D5 : FITC-mc-1Stat3; D6: Ctr-mc or linear 95 bp; D7: biotinylated Ctr-mc; D8: FITC-Ctr-mc. Nick is represented by an interruption within nucleotide sequence in either top or bottom strand giving rise to an overlapped double-stranded region allowing higher DNA bendability and hence efficient circularization reaction for minicircle production. With duplexes D1 to D5, the nucleotides shown in bold indicate the presence of one (D1, D4), two (D2) and three (D3) customized STAT3 binding sequence. With the duplexes D4 and D7, (◆) denotes the presence of a biotin residue attached to position 5 of thymine; with the duplexes D5 and D8, (●) indicates the presence of a fluorescein residue (FITC) attached to position 5 of thymine ring by a 6-carbons spacer arm.

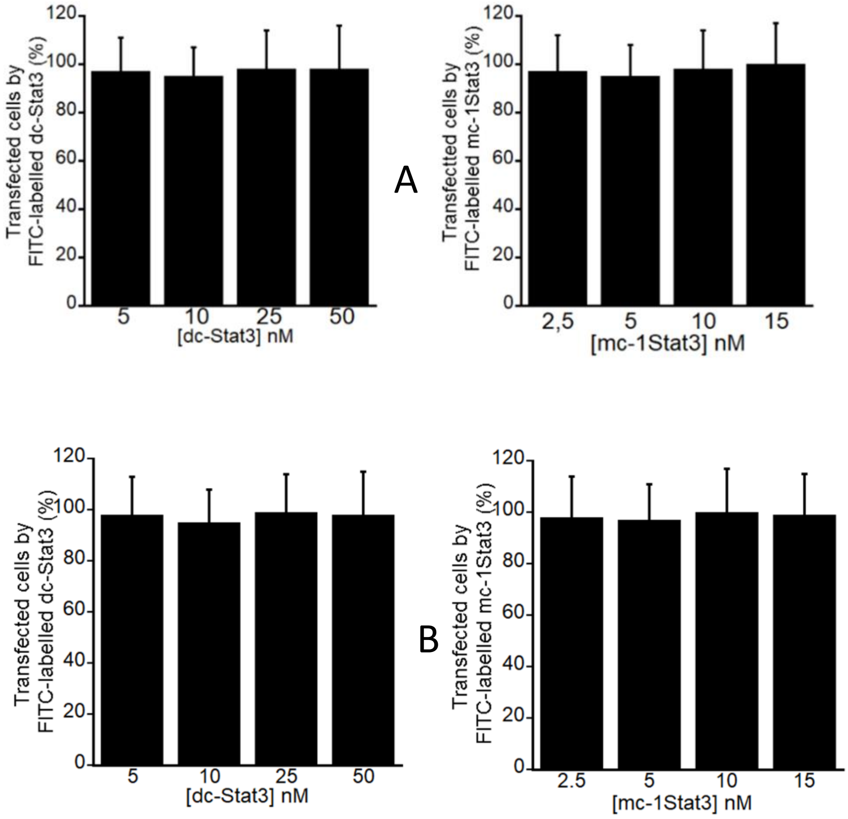

Figure S6. Cell transfection efficiency is independent of STAT3 linear and MC decoy concentration. MDA-MB-231 (A) or 4T1 (B) cells were transfected in the presence of increasing concentration of fluorescent-labelled ODN formulated with lipofectamine 3000 and 24 hours later the percentage of positive fluorescent cells was determined by flow cytometry.

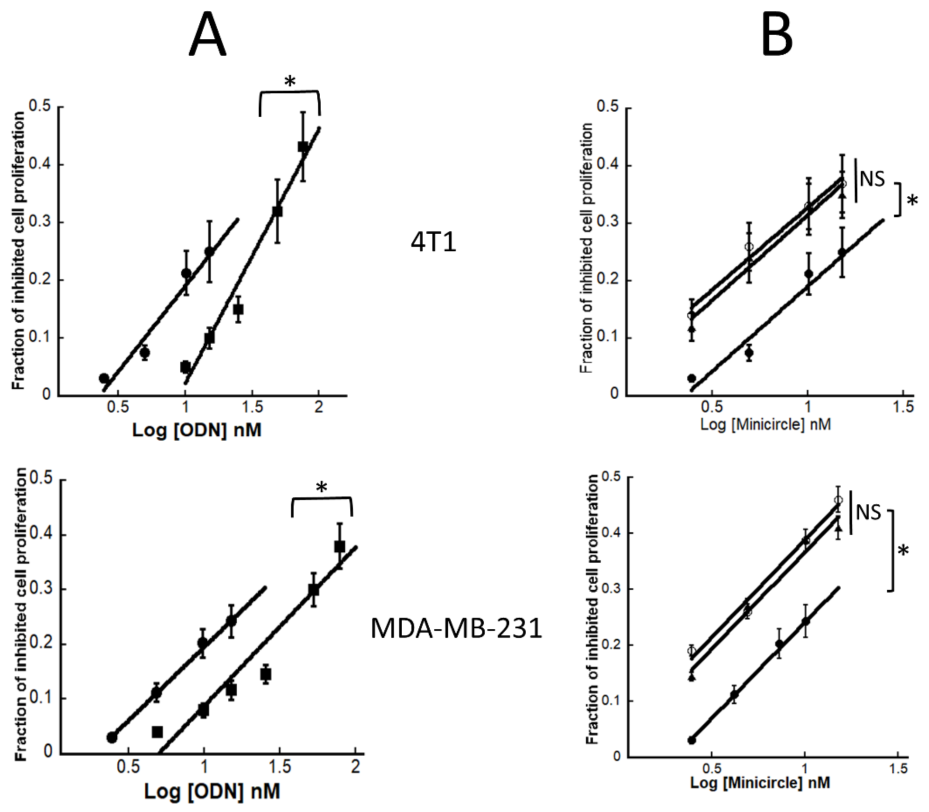

Figure S7. Linearization of cell proliferation dose-response curve. (A) Comparison of TNBC cells treatment with dc-Stat3 versus mc-1Stat3; (B) TNBC cells treatment with MC containing one, two or three STAT3 binding sites. (■) dc-Stat3; (●) mc-1Stat3 (▲) mc-2Stat3; (○) mc-3Stat3. Linearization was performed by plotting the fraction of inhibited cell proliferation as a function of logarithm base 10 of nucleic concentration (1). The concentration of each nucleic acids that induced 20% of inhibition (IC20) was then calculated from the corresponding dose response curve. All the data are expressed as the means ±SD (*$P < 0.05$; ** $P < 0.01$; *** $P < 0.001$; NS, not significant).

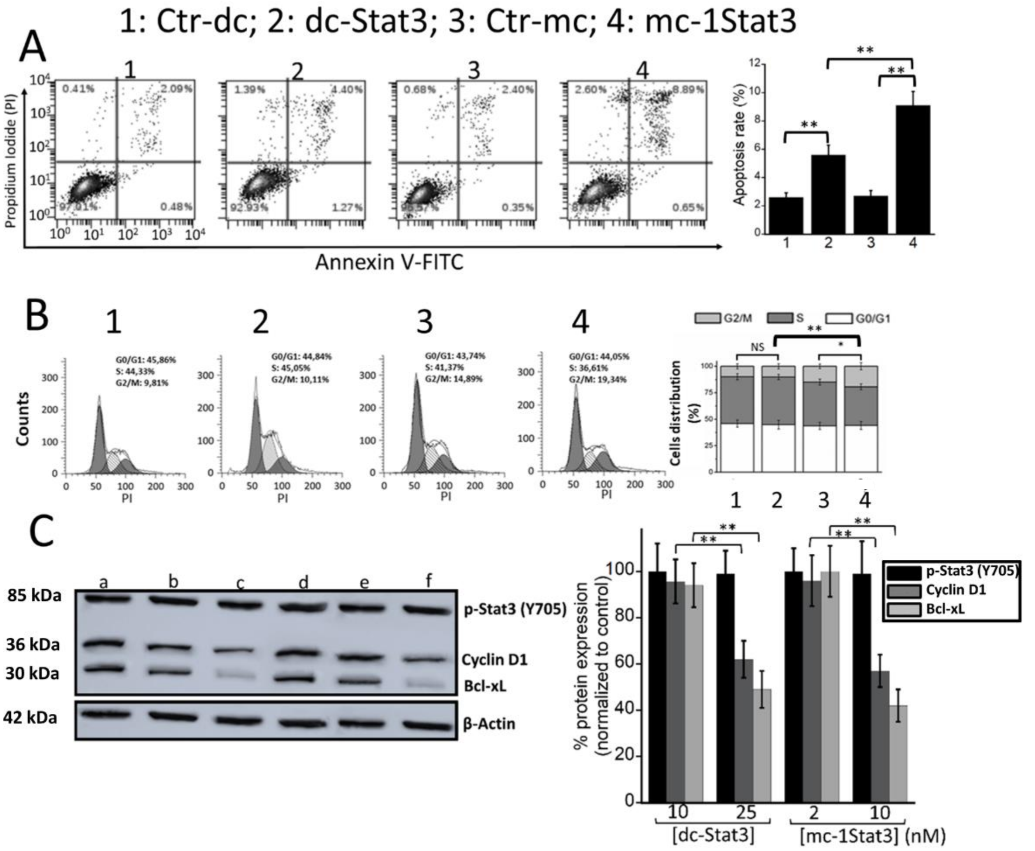

Figure S8. mc-1Stat3 triggers apoptosis, induces G2/M phase cell cycle arrest and inhibits Stat3-dependent gene expression more efficiently than dc-Stat3 in 4T1 cells: (A) Detection of apoptotic 4T1 cells by flow cytometry analysis using Annexin V-FITC and propidium iodide: cells were transfected with 15 nM of mc-1Stat3, dc-Stat3, Ctr-mc or Ctr-dc and then stained with annexin V-FITC and propidium iodide 48 h later. Result of apoptosis assays is shown on the right side as histogram, 4T1 cells positive for both Annexin V and PI being considered as apoptotic cells. (B) 4T1 cell cycle progression analysis: 4T1 cells were transfected as in (A) and cell cycle distribution was then assessed using flow cytometry. Representative results of the cell cycle analyses and histogram of cell cycle distribution are shown on the left and on the right, respectively. (C) Expression level of p-STAT3 (Y705), Bcl-xL, Cyclin-D1 following 4T1 cells treatment with mc-1Stat3 and dc-Stat3. 4T1 were treated with dc-Stat3 or mc-1Stat3 and total cell extract was obtained 48 h later after cell lysis. Protein expression was assessed by western blot as indicated on the left side of Figure C. Lane (a): Ctr-dc; lane (b) and (c): dc-Stat3, 10 and 25 nM, respectively; lane (d): Ctr-mc; lane (e) and (f): mc-1Stat3, 2 and 10 nM, respectively. β-actin protein was used as internal loading control. On the right side of Figure C is shown the quantified expression levels of proteins as a function of the nature and dose of decoy ODNs as indicated. Results presented are representative of three independent experiments performed in triplicates. All the data are expressed as the means ±SD of three independent experiments (*$P < 0.05$; ** $P < 0.01$; *** $P < 0.001$; NS, not significant).

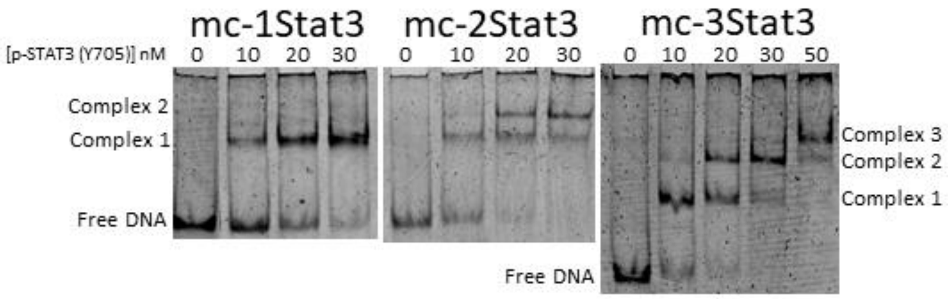

Figure S9. Titration of STAT3 by decoy MC increases as a function of the number of DNA binding sites. Each type of minicircle was incubated with increasing concentrations of p-STAT3 (Y705) protein as indicated and the reaction mixture analyzed by electrophoretic mobility shift assay (EMSA). Free DNA indicates migration of MC in the absence of protein. The shifted band named complex 1, complex 2 and complex 3 corresponds to gel-shifted complex containing one, two and three proteins per minicircle, respectively. Near complete saturation of the binding sites by STAT3 is reached for the three MCs as shown by the presence of a major shifted band. The binding assay and the DNA binding activity of p-STAT3 (Y705) was performed by EMSA as described (2). Recombinant histidine-tagged p-STAT3 (Y705) protein was produced using plasmid pet28a STAT3 βTC vsv his tag (a kind gift from Prof. Thorsten Berg, University of Leipzig, Germany) and purified as previously described (3).

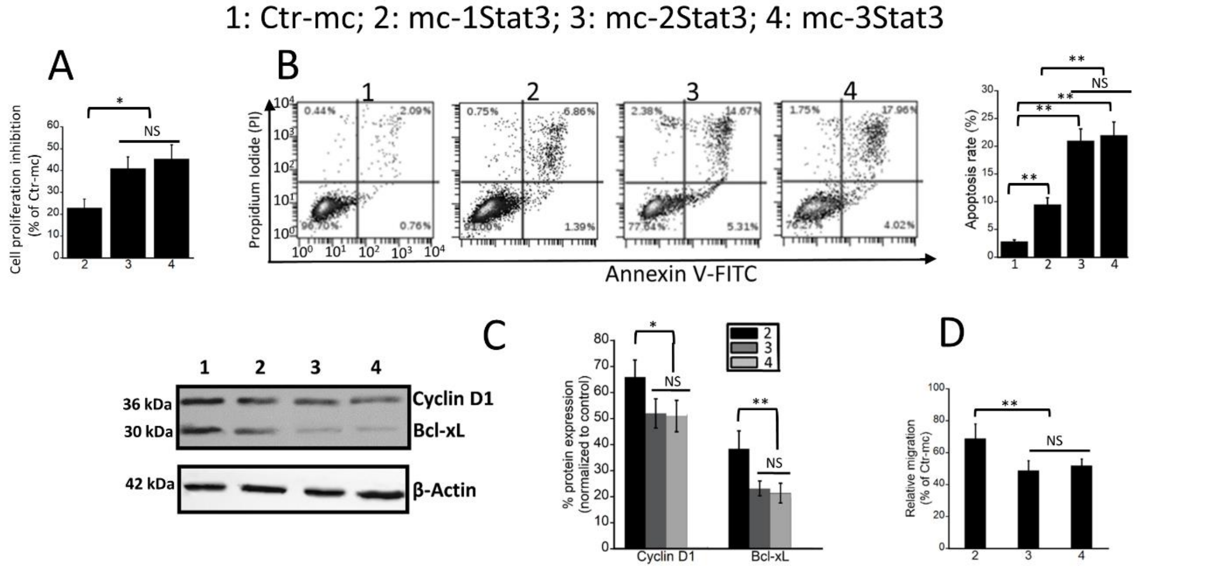

Figure S10. Effect of minicircle STAT3 binding sites number on 4T1 cellular activity.

A) Comparative proliferation evaluation of 4T1 cells treated with mc-1Stat3, mc-2Stat3, mc-3Stat3 (10 nM); cell survival was determined 48 h after using Alamar Blue assay. B) Detection of apoptotic 4T1 cells by flow cytometry analysis using Annexin V-FITC and propidium iodide. 4T1 cells were treated with 10 nM of mc-1Stat3, mc-2Stat3, mc-3Stat3 and Ctr-mc; 48 h later, the level of apoptosis was evaluated using the AnnexinV/PI dual-labeling technique, as determined by flow cytometry. Quantitative results of apoptosis assays is shown on the right side as histogram. C) Expression level of Bcl-xL and Cyclin-D1 following 4T1 cells treatment with mc-1Stat3, mc-2Stat3 and mc-3Stat3**.** Western blot assay of 4T1 treated with minicircles bearing one to three STAT3 binding sites and with Ctr-mc at 10 nM concentration. Cell lysates were immunoblotted for the indicated proteins. β-actin was used as a loading control. D) Migration inhibition of 4T1 cells induced by STAT3 MCs. 4T1 cells were first treated with 10 nM concentration of minicircle and 24 hours post-treatment, migration was determined using Transwell migration assay. After 6 hours, the migratory cells were counted and the quantification is shown on histogram analysis. Results presented are representative of three independent experiments performed in triplicates. All the data are expressed as the means ±SD of three independent experiments, (* $P < 0.05$; ** $P < 0.01$; *** $P < 0.001$; NS, not significant).

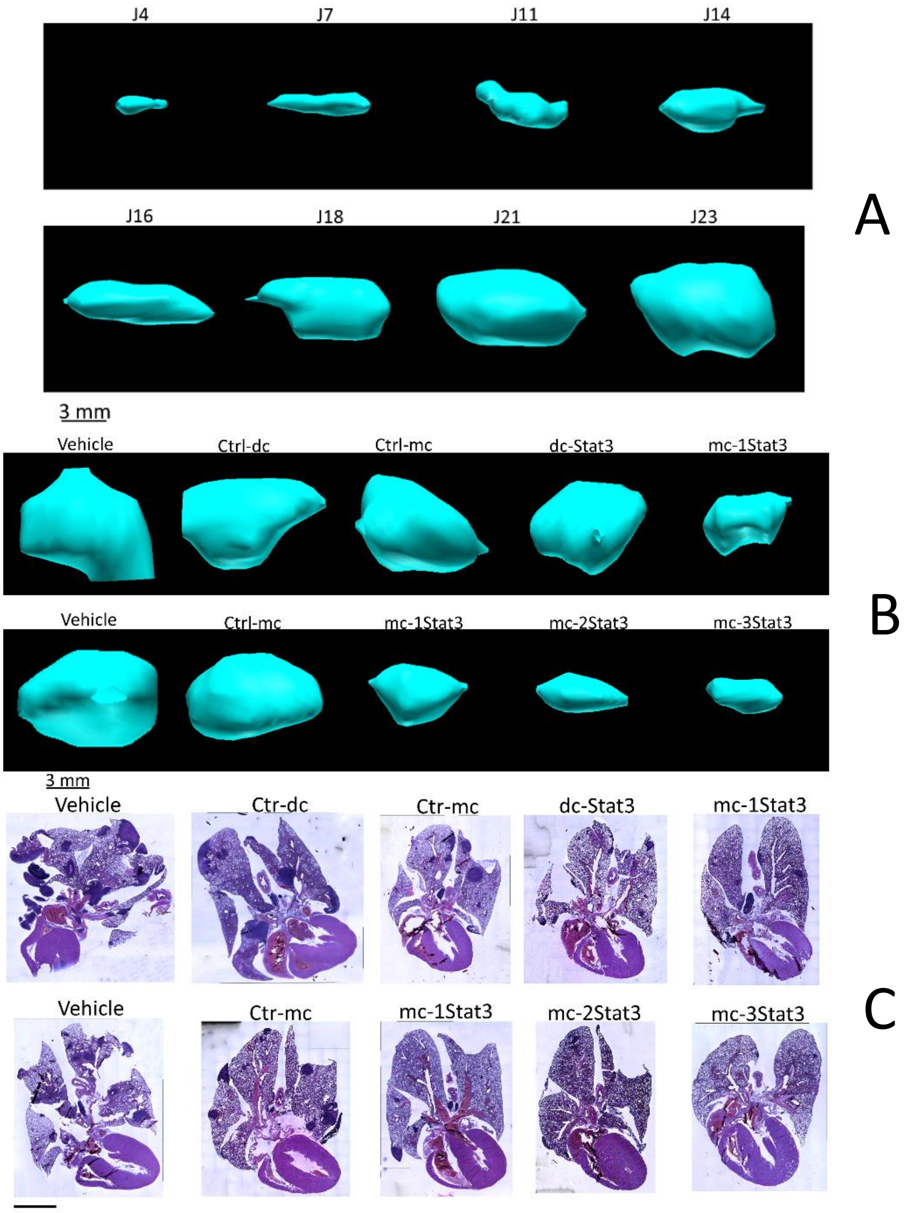

Figure S11. Representation of primary 4T1 tumor 3D reconstruction of ultrasound imaging data and H&E staining of lung section illustrating *in vivo* data obtained with or without treatment. Examples showing A) the time-dependent change in tumor size for one mice belonging to the vehicle group, B) the mice group-dependent primary tumor size as a function of treatment at 26 days following 4T1 cells inoculation within mammary fat pad, C) the mice group-dependent lungs sections used to visualize metastatic foci (dark purple) as a function of treatment (Scale bar = 1 cm). Vevo Labs and ImageJ softwares were used for production of 3D images following tumor volume measurement and surface area measurement of metastatic foci, respectively.

Reference:

1) Chou, T., C. (2006). Theoretical basis experimental design and computerized simulation of synergism and antagonism in drug combination studies. Pharmacological reviews, *58*, 621-681.
2) Thibault, T., Degrouard, J., Baril, P., Pichon, C., Midoux, P., Malinge, J.-M., (2017). Production of DNA minicircles less than 250 base pairs through a novel concentrated DNA circularization assay enabling minicircle design with NF-κB inhibition activity. Nucleic Acids Res. *45*, e26.
3) Becker, S., Corthals, G.,L., Aebersold, R., Groner, B., Müller, C., W., (1988). Expression of a tyrosine phosphorylated, DNA binding Stat3beta dimer in bacteria. FEBS Lett. *441*, 141-147.
