# Supplementary material for: DNA minicircles as novel STAT3 decoy oligodeoxynucleotides endowed with anticancer activity in triple-negative breast cancer
Source: Mol Ther Nucleic Acids. 2022 Jun 22;29:162–75. doi: 10.1016/j.omtn.2022.06.012 (PMC9263874; doi:10.1016/j.omtn.2022.06.012)
Supplement: Document S2. Article plus supplemental information [file mmc2.pdf]

Molecular Therapy
Nucleic Acids
Original Article

DNA minicircles as novel STAT3 decoy oligodeoxynucleotides endowed with anticancer activity in triple-negative breast cancer

Geoffrey Casas,[1,2] Federico Perche,[1] Patrick Midoux,[1] Chantal Pichon,[1] and Jean-Marc Malinge[1]

[1]Centre de Biophysique Moléculaire, UPR 4301 CNRS, Affiliated with the University of Orléans and INSERM, Rue Charles Sadron, CS-80054, 45071 Orléans Cedex 02, France

Decoy technology is a versatile and specific DNA oligonucleotide-based targeting strategy of pathogenic transcription factors (TFs). Chemical modifications of linear decoy oligonucleotides have been made to decrease nuclease sensitivity because of the presence of free ends but at the cost of new limitations that affect their use as therapeutic drugs. Although a short DNA minicircle is a phosphodiester nucleic acid without free ends, its potential therapeutic activity as a TF decoy oligonucleotide has not yet been investigated. Here we describe the *in vitro* and *in vivo* activity of formulated 95-bp minicircles bearing one or several STAT3 binding sequences in triple-negative breast cancer (TNBC). Minicircles bearing one STAT3 binding site interacted specifically with the active form of STAT3 and inhibited proliferation, induced apoptosis, slowed down cell cycle progression, and decreased STAT3 target gene expression in human and murine TNBC cells. Intratumoral injection of STAT3 minicircles inhibited tumor growth and metastasis in a murine model of TNBC. Increasing the number of STAT3 binding sites resulted in improved anticancer activity, opening the way for a TF multi-targeting strategy. Our data provide the first demonstration of minicircles acting as STAT3 decoys and show that they could be an effective therapeutic drug for TNBC treatment.

INTRODUCTION

Oligonucleotide-based molecular therapies are increasingly recognized as promising treatments for several human diseases because of their ability to specifically control target gene expression. This family of therapeutic molecules encompasses various types of oligonucleotide structures and compositions, such as small interfering RNA and antisense oligonucleotides, which target specific mRNA or DNA decoys for direct protein targeting.[1] Several antisense oligonucleotides[2] and three small interfering RNA (siRNA) oligonucleotides[3] have been approved by the US Food and Drug Administration (FDA). This is not the case for decoy oligonucleotides despite the fact that the decoy strategy is a unique and versatile therapeutic approach used to target relevant transcription factors (TFs), notably those that are not readily druggable with small-molecule inhibitors.

Every TF is capable of sequence-specific DNA binding activity in the promoter region of target genes for transcription regulation. This binding activity is exploited in the decoy strategy to trap the target TF by using a short double-stranded oligodeoxynucleotide (ODN) containing a TF binding site. When delivered into cells, the decoy ODN efficiently inhibits the target TF by competitive binding activity with its genomic binding sites, enabling subsequent control of pathogenic downstream TF target genes.[4,5] Because a growing number of TFs are directly involved in diseases such as cancer, cardiovascular diseases, and musculoskeletal disorders,[6,7] a wealth of decoy ODNs have been tested *in vitro* and in pre-clinical animal studies, showing the versatility of this small nucleic acid strategy in targeting TFs.[4] Only four TF-based decoys have advanced to clinical trials. They include decoy ODNs for targeting of E2F to prevent bypass vein graft failure,[8] EGR1 to treat surgical pain,[9] nuclear factor κB (NF-κB) to reduce discogenic low back pain (ClinicalTrials.gov: NCT03263611), and signal transduction and activator of transcription 3 (STAT3) for head and neck cancer treatment.[10]

A key limitation of the application of decoys as therapeutic agents is their rapid degradation in the cellular environment and in serum. Indeed, natural phosphodiester backbone DNA molecules are subject to rapid degradation through the action of nuclease attack.[11,12] To improve the biostability of decoys with free ends, phosphorothioation at the internucleotide linkage provided the first generation of decoys with higher biostability.[13] However, several studies have reported inactivity because of limited stability.[10,14] The effects produced by several types of nucleotide chemical modifications are unexpected and include important drawbacks in terms of TF affinity, ODN thermal denaturation, and *in vivo* toxic effects, leading to restrain the chemical modifications as much as possible outside of the TF binding sequence.[15] Because free ends are well known to be responsible for

Received 13 January 2022; accepted 15 June 2022;
https://doi.org/10.1016/j.omtn.2022.06.012.

[2]Present address: Agro-Bio, 2 All. de la Chavannerie, 45,240 La Ferté-Saint-Aubin, France

Correspondence: Jean-Marc Malinge, Centre de Biophysique Moléculaire, UPR 4301 CNRS, Rue Charles Sadron, CS-80054, 45071 Orléans, Cedex 02, France.
E-mail: malinge@cnrs-orleans.fr
Correspondence: Chantal Pichon, Centre de Biophysique Moléculaire, UPR 4301 CNRS, Rue Charles Sadron, CS-80054, 45071 Orléans, Cedex 02, France.
E-mail: chantal.pichon@cnrs.fr

 © 2022 The Authors.
This is an open access article under the CC BY license (http://creativecommons.org/licenses/by/4.0/).

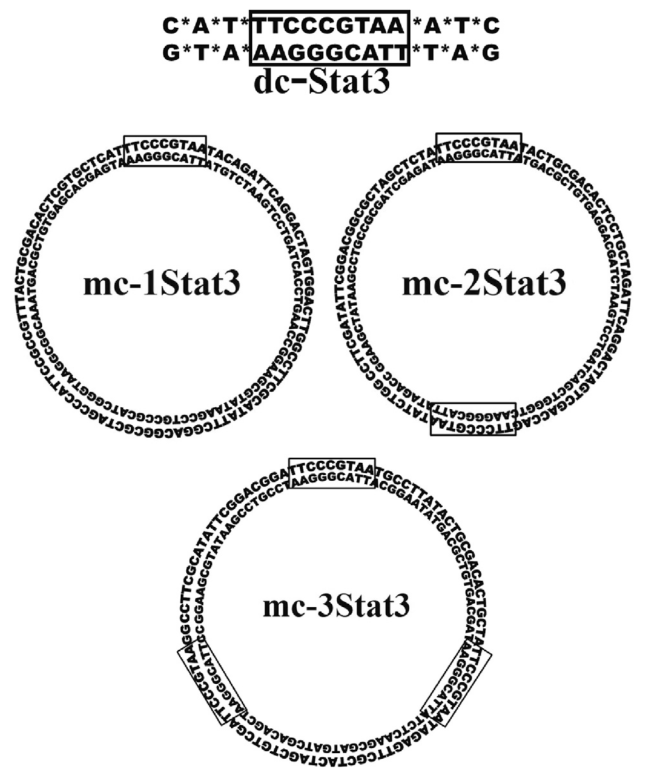

Figure 1. ODNs used as STAT3 molecular decoys in this study

For linear and MC ODNs, a rectangular frame indicates the presence and position of the specific STAT3 binding sequence. All MCs have the same length (95 bp), and the name of their respective abbreviation is indicated in each MC schematic; for instance, mc-1Stat3 indicates that the MC is carrying a single STAT3 binding site. Asterisks indicate phosphorothioate linkage present in linear decoy ODNs directed against STAT3. The corresponding linear and MC nonspecific control ODNs are abbreviated as Ctr-dc and Ctr-mc, respectively (see supplemental information).

decoy exonuclease degradation, closure of DNA strands has been performed to yield a phosphodiester decoy with a dumbbell-shaped structure that exhibits complete resistance to exonuclease.[11] However, the DNA single-stranded nature of the loop linking the 5′ and 3′ ends of both strands of a duplex decoy has been shown to be sensitive to single-stranded endonucleases.[16] Therefore, the DNA single-stranded loop was replaced by a chemical linkage at both decoy termini[10,17,18] or at one terminus with an unlinked chemical chain-terminating ODN at the opposite side,[19] giving more *in vivo* stability to this novel form of decoys that were used to inhibit different TFs. The presence of the chemical linkage does not induce toxicity after intravenous administration in nude mice,[20] but a phase I clinical trial has not yet been initiated, to our knowledge, to confirm a lack of toxicity. It will be of great interest to design a new decoy devoid of chemical modifications, free ends, and nucleotide loops.

Double-stranded minicircles (MCs) 300—1,000 bp in length recently entered the field of gene therapy with a better delivery efficiency than conventional exogenous plasmid-based vectors used as nucleic acid therapeutic agents.[21] Considering the various technical limitations that impair improvement of TF decoy inhibitors, we recently proposed that DNA MCs could have several biological properties that are still lacking with linear decoys with or without free ends. To design MCs with potential decoy activity, we decided to shorten MC size. A main hurdle toward this goal was the absence of a method for production of MCs of a size down to 250 bp. To circumvent this problem, we used a versatile and quantitative production method of MCs having a size down to 250 bp with the possibility to incorporate site-specific customized sequences and chemical functionalization. We also showed that relaxed 95-bp MCs containing the NF-κB sequence are endowed with the capacity to avidly bind the TF NF-κB and to inhibit its transcriptional activity.[22]

In the current study, our aim was to determine whether MCs could be used as efficient decoy ODNs for TF targeting with therapeutic applications. We chose STAT3 as a target, given that this oncogenic TF has been targeted with the previous generation of decoy ODNs and is a well known but undruggable target in cancer therapy. Indeed, STAT3 is a main oncogenic TF whose activation contributes to expression of target genes implicated in tumor cell proliferation, survival, tumor invasion, angiogenesis, metastasis, and immunosuppression. STAT3 is persistently activated in cancer cells, leading to phosphorylation of tyrosine 705 by Janus kinases.[23–25] STAT3 is aberrantly activated in the majority of breast cancers, including triple-negative breast cancer (TNBC).[26,27] Recent data provide evidence that increased STAT3 expression occurs in young individuals with TNBC[28] and that STAT3 activation is correlated with worse survival.[29] Because TNBC treatment cannot benefit from hormonal therapy because of a lack of targetable receptors, there is an urgent need to develop molecularly targeted treatments for TNBC. Here we designed relaxed 95-bp MCs bearing one or several STAT3 binding sites and assessed their biological activity. We show that formulated STAT3 MCs enabled specific STAT3 targeting that resulted in proliferation inhibition, apoptosis induction, slowing of cell cycle progression, and a decrease in STAT3 target gene expression. Intratumoral treatment with formulated STAT3 MCs led to tumor growth inhibition in an orthotopic TNBC mouse model with decreased expression of STAT3 target genes in the tumor, along with a reduction in lung metastasis. Increasing the number of STAT3 binding sites in MCs resulted in improved anticancer activity. A comparative study of STAT3 MCs and parent phosphorothioated STAT3 linear decoys indicated significantly more potent MC anticancer activity, which correlated with greater cellular biostability. Our data demonstrate, for the first time, that MCs act as new structural STAT3 decoy ODNs in TNBC.

RESULTS

MC intracellular delivery, biostability, and target interaction

Our aim was first to determine whether DNA MCs designed to target STAT3 performs cellular and molecular activities required for decoy activity while making a comparison with the parent phosphorothioated decoy ODNs with free ends used previously to inhibit STAT3.[10] The sequence compositions of 95-bp MCs containing one STAT3 binding site (mc-1Stat3) and that of parent linear STAT3 decoy ODNs (dc-Stat3) are shown in Figure 1.

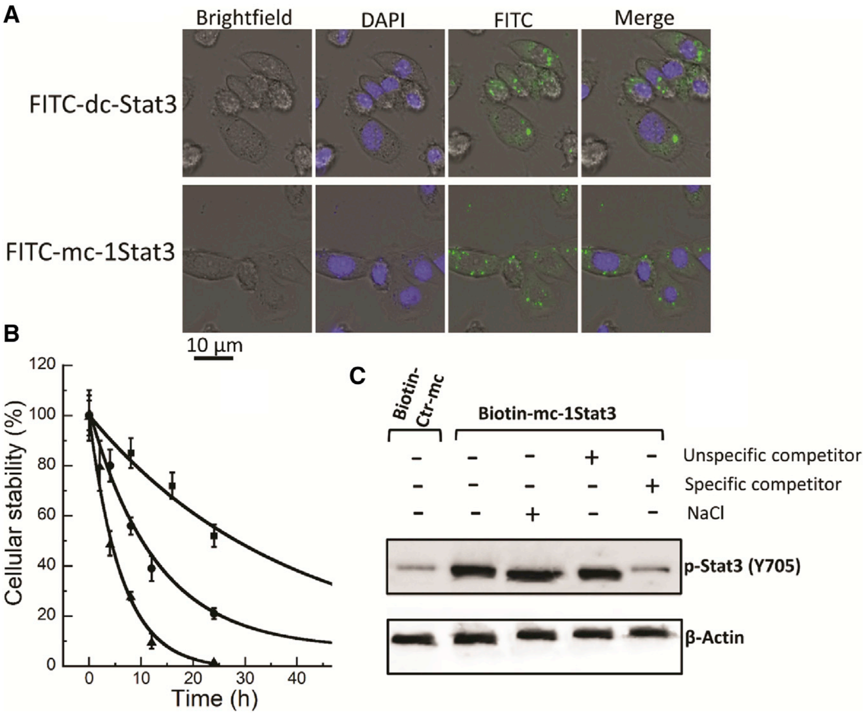

Figure 2. MC intracellular uptake, biostability, and specific target interaction

(A) Comparative uptake of fluorescein-labeled dc-Stat3 and mc-1Stat3 by MDA-MB-231 cells. 6 h after transfection with Lipofectamine 3000/ODN complexes (15 nM), cells were analyzed by confocal laser-scanning microscopy to assess the intracellular localization of FITC-labeled linear decoy dc-Stat3 and MC mc-1Stat3 (green). Nuclei were stained with DAPI (blue). (B) DNA MCs have higher biostability than linear decoys in MDA-MB-231 cellular extract. Phosphorothioated (dc-Stat3), phosphodiester linear STAT3 decoy ODNs of 15 bp and mc-1Stat3 MCs were incubated at 37°C with cell extract as a function of time. Each time point corresponds to the starting amount of nucleic acids as analyzed by gel electrophoresis (Figure S4). The curves represent the fit to a single exponential. Data points and error bars represent the average and SD from at least three experiments, respectively. Shown are mc1-Stat3 (■), dc-Sta3 (●), and linear phosphodiester STAT3 decoys (▲). (C) A pull-down assay shows the specific interaction of mc-1Stat3 with p-STAT3 (Y705) in MDA-MB-231 cell extract. A biotinylated version of mc-1Stat3 or Ctr-mc (200 ng) was incubated in nuclear extract (25 µg). To the nuclear extract containing the biotinylated mc-1Stat3, an excess of non-biotinylated Ctr-dc (nonspecific competitor) or dc-Stat3 (specific competitor) was added, or the ionic strength was increased to 150 mM NaCl, as indicated. The nucleoprotein complex was then captured by adding streptavidin magnetic beads. The complexes were analyzed by western blotting using anti-p-STAT3 (Y705) antibodies. β-Actin input levels served as loading controls.

We first determined whether mc-1Stat3 and dc-Stat3 are internalized in MDA-MB-231 cells using mc-1Stat3 and dc-Stat3 labeled with one fluorophore (fluorescein) and formulated with the commercial cationic lipid Lipofectamine 3000 as a transfection agent. We verified that each type of ODN is totally complexed in the presence of this lipid by gel retardation assay (Figure S1). As shown in Figure 2A, images of cells incubated with labeled mc-1Stat3 or dc-Stat3 formulated with Lipofectamine 3000 were very similar, suggesting that both ODNs are successfully taken up into cells. Cells transfected with mc-1Stat3 or dc-Stat3 exhibited a few fluorescent spots and diffuse fluorescence staining throughout the cytoplasm that likely correspond to complexed ODNs in endosomes and to released ODN homogeneously distributed in the cytosol of most of the cells, respectively. The nucleus compartment was slightly fluorescent, suggesting that dc-Stat3 and mc-1Stat3 are retained in the cytosol. The uptake of both ODNs was also measured by cytometry. Fluorescent dc-Stat3 and mc-1Stat3 were taken up at similar levels in MDA-MB-231 cells (Figure S2). We also compared the delivery of both ODNs in another TNBC cell line; as shown in Figure S3, both fluorescent ODNs were delivered similarly into murine 4T1 cells, as observed for MDA-MB-231. Our data indicate that mc-1Stat3 and dc-1Stat3 have similar cellular delivery and intracellular location, demonstrating that the shape and size of mc-1Stat3 do not affect its uptake and intracellular trafficking compared with the parent linear decoy dc-Stat3.

An important parameter for decoy activity is the limited ODN biostability in the cellular environment because of the presence of nucleases. Because we did not yet verify whether 95-bp DNA MCs have sufficient stability in cells for decoy activity, we next compared the stability of mc-1Stat3 and dc-Stat3 at 37°C in MDA-MB-231 cell extract as a function of time. As shown in Figure 2B, the amount of nucleic acids was determined as a function of incubation time from the electrophoresis experiments shown in Figure S4. We found that mc-1Stat3 exhibits a half-life of 29 ± 3.5 h, whereas linear dc-Stat3 and its phosphodiester counterpart have half-lives of 12 ± 1.2 and 4.3 ± 0.7 h, respectively. Our data show, for the first time, that chemically unmodified MCs afford significantly increased stability of the phosphodiester backbone, resulting in a half-life increase of about two times as compared with first-generation phosphorothioated dc-Stat3 decoys. Thus, removal of free ends through circularization of linear ODNs is an effective strategy to substantially increase resistance to intracellular nuclease digestion.

We next determined whether cellular STAT3 protein could be trapped by mc-1Stat3, which is a pre-requisite for proper decoy activity. Toward this goal, we performed a pull-down assay using mc-1Stat3 and Ctr-mc containing a site-specifically placed biotin residue (Figure S5) to verify the direct and specific interaction with the phosphorylated form of STAT3 (i.e., p-STAT3 [Y705]), known to exhibit sequence-specific DNA binding activity. As shown in lane 2 of Figure 2C, biotinylated mc-1Stat3 formed a complex with p-STAT3, as

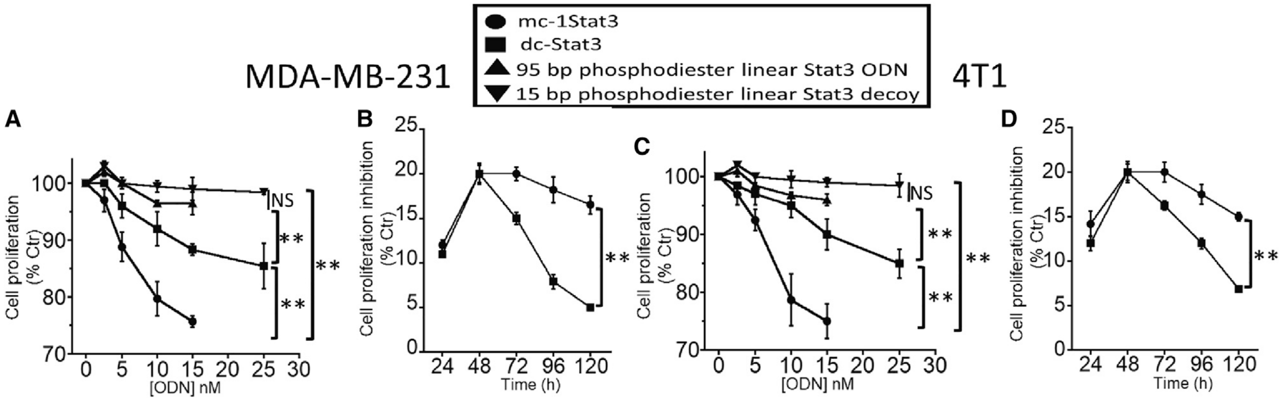

Figure 3. Proliferation inhibition of MDA-MB-231 and 4T1 cells induced by mc-1Stat3 is more efficient and time persistent compared with dc-Stat3

(A and C) Cell proliferation dose-response curve of mc-1Stat3, dc-Stat3, and linear phosphodiester STAT3 ODNs 15 and 95 bp in length with MDA-MB-231 and 4T1 cells. Cells were treated with formulated ODNs at concentrations ranging from 0–25 nM as indicated, and then cell survival was determined by alamarBlue assay 48 h after treatment. (B and D) Time-dependent proliferation inhibition of mc-1Stat3 and dc-Stat3. Cells were treated with mc-1Stat3 and dc-Stat3 at a final concentration of 10 nM and 25 nM, respectively. Cell proliferation was determined as a function of time. *In vitro* proliferation inhibition for each type of specific STAT3 decoy ODN was normalized to the corresponding nonspecific control ODN. Errors bars indicate SD of at least three independent experiments (*p < 0.05; **p < 0.01; ***p < 0.001; NS, not significant).

deduced from the large band corresponding to immunodetected p-STAT3; this band was faint with nonspecific biotinylated Ctr-mc substrate (lane 1). A competition experiment of p-STAT3 binding to biotinylated mc-1Stat3 was carried out with linear nonspecific Ctr-dc (lane 4) and specific dc-Stat3 (lane 5) decoys and showed that the nucleoprotein complex was only disrupted with specific STAT3 competitors. This specific p-STAT3/mc-1Stat3 interaction was resistant to a salt concentration increase (lane 3), which is in agreement with an efficient and specific STAT3 trapping capacity of mc-1Stat3.

Our data from ODN cellular uptake, stability, and STAT3 binding experiments allow us to conclude that mc-1Stat3 MCs possess appropriate properties to act as STAT3 decoys.

STAT3 MCs inhibit TNBC cancer cell proliferation more efficiently than STAT3 linear DNA decoys

The effect on cell proliferation induced by mc-1Stat3 and linear dc-Stat3 was compared using MDA-MB-231 and 4T1 cells, which were incubated with increasing concentrations of ODN/Lipofectamine 3000 complexes. Cell proliferation was determined 48 h after treatment using an alamarBlue assay. Because specific decoy ODNs and their respective control ODN were taken up equally by TNBC cells at a fixed concentration (Figure S2), we expressed the cell proliferation data as the percentage of the respective control ODN.The percentage of transfected TNBC cells was identical across all tested ODN concentrations, indicating that dose-response proliferation curves are independent of the percentage of transfected cells (Figure S6).

As shown in Figures 3A and 3C, MDA-MB-231 and 4T1 cells treated with mc-1Stat3 show a dose-dependent reduction in cell proliferation. This result shows, for the first time, that decoy ODNs as STAT3 MCs are endowed with TNBC cancer cell anti-proliferative activity. We also observed for both TNBC cell lines that linear dc-Stat3 is less efficient in inhibiting cell proliferation compared with mc-1Stat3. Linearization of the dose-effect curve allowed us to determine that mc-1Stat3 inhibits cell proliferation with an IC20 of 11 nM, whereas that of dc-Stat3 is 25 nM, indicating that mc-1Stat3 is significantly more potent by 2-fold compared with dc-Stat3 ($p < 0.05$) (Figure S7). The linear form of phosphodiester 15 and 95-bp ODNs containing a STAT3 binding site was found to be nearly inactive in inhibiting cell viability, likely as a consequence of high susceptibility to degradation in TNBC cells. As shown in Figures 3B and 3D, the time-dependent cell proliferation inhibition induced by mc-1Stat3 and dc-Stat3 at 10 nM reveals that mc-1Stat3 affords a longer time of antiproliferative activity compared with dc-Stat3 by nearly 2- and 3-fold in MDA-MB-231 and 4T1 cells, respectively ($p < 0.001$). These results show that STAT3 decoy MCs exhibit antiproliferative activity in a dose and time-dependent manner in TNBC cells.

STAT3 MCs induce apoptosis and cell cycle arrest and decrease STAT3 target gene expression in TNBC cells

To follow up, we determined whether TNBC cells undergo apoptosis differentially when treated with mc-1Stat3 versus dc-Stat3 used at 15 nM. The level of apoptosis was determined using the Annexin V-fluorescein isothiocyanate (FITC)/PI dual labeling technique by flow cytometry 48 h after treatment. As shown in Figures 4A and 4B, the apoptosis rates induced in MDA-MB-231 cells by mc-1Stat3 and dc-Stat3 are 18% ± 2.7% and 8% ± 1.3%, respectively. This reveals that STAT3 MC is significantly more efficient in inducing apoptosis compared with linear decoys ($p < 0.001$). We next analyzed the cell cycle distribution of MDA-MB-231 cells treated under the same conditions as above using flow cytometry. As shown in Figures 4C and 4D, the number of cells in G2/M phase increased significantly by 50% after treatment with mc-1Stat3 compared with Ctr-mc ($p < 0.001$). Blockade of G2/M phase was also observed upon

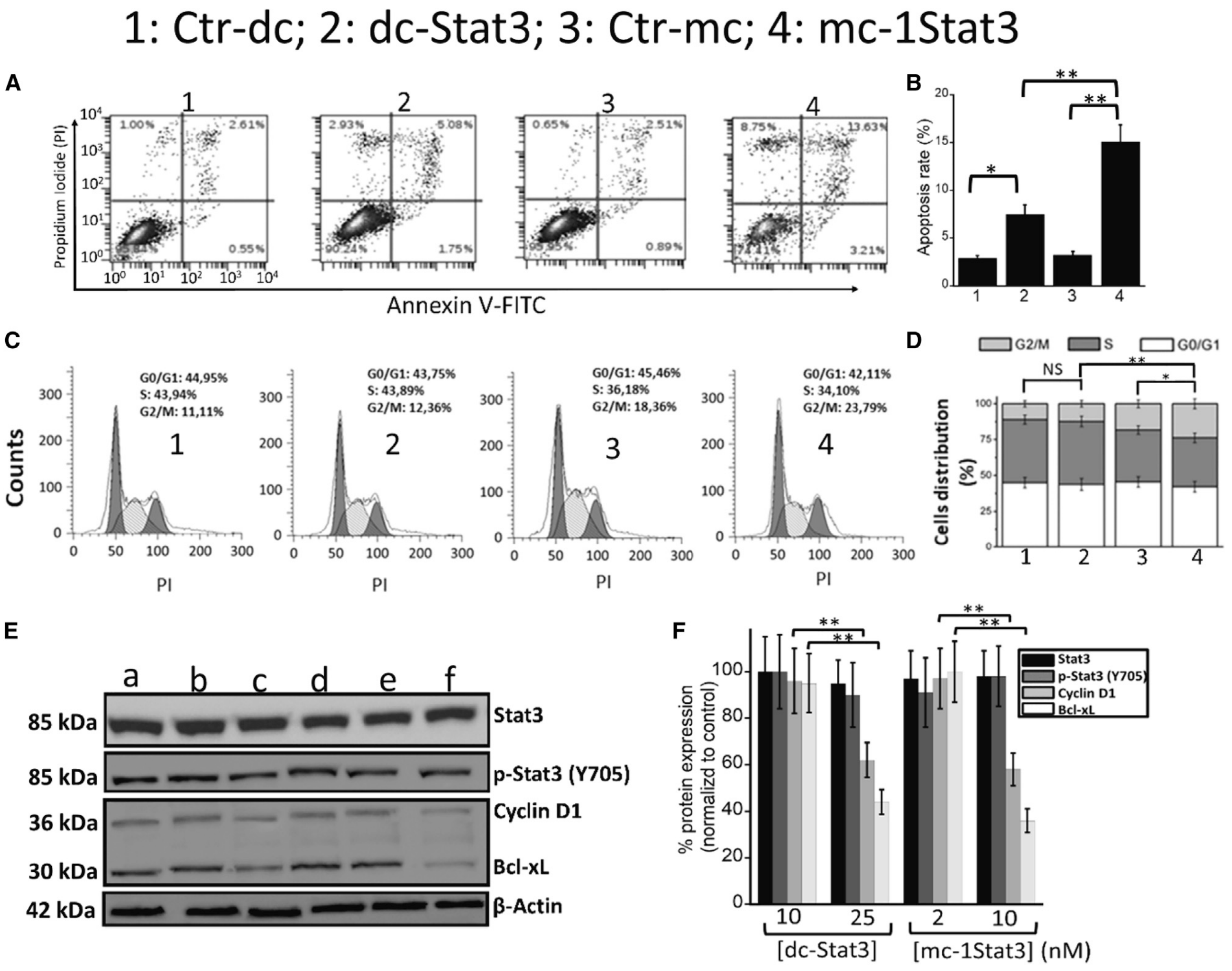

Figure 4. mc-1Stat3 triggers apoptosis, induces G2/M phase cell cycle arrest, and inhibits Stat3-dependent gene expression more efficiently than dc-Stat3 in MDA-MB-231 cells

(A and B) Detection of apoptotic MDA-MB-231 cells by flow cytometry analysis using Annexin V-FITC and propidium iodide. Cells were transfected with 15 nM mc-1Stat3, dc-Stat3, Ctr-mc, or Ctr-dc and stained with Annexin V-FITC and propidium iodide 48 h later. The results of the apoptosis assays are shown as histograms (B), with MDA-MB-231 cells positive for Annexin V and PI considered apoptotic cells. (C and D) MDA-MB-231 cell cycle progression analysis after various ODN treatments as indicated. MDA-MB-231 cells were transfected as in (A), and cell cycle distribution was assessed using flow cytometry (C) Histograms of cell cycle distribution are shown in (D). (E and F) Expression level of STAT3, p-STAT3 (Y705), Bcl-xL, and Cyclin D1 after MDA-MB-231 cell treatment with mc-1Stat3 and dc-Stat3. MDA-MB-231 cells were treated with dc-Stat3 or mc-1Stat3, and total cell extract was obtained 48 h later after cell lysis. Protein expression was assessed by western blot (E). Lane a: Ctr-dc; lanes b and c: dc-Stat3, 10 and 25 nM, respectively; lane d: Ctr-mc; lanes (e) and (f): mc-1Stat3, 2 and 10 nM, respectively. β-Actin protein was used as an internal loading control. The quantified expression levels of proteins as a function of the nature and dose of decoy ODNs is shown in (F). Results presented are representative of three independent experiments performed in triplicates. All data are expressed as the means ± SD of three independent experiments (*$p < 0.05$; **$p < 0.01$; ***$p < 0.001$).

treatment of the cells with dc-Stat3, but the difference was not statistically significant compared with Ctr-dc. We examined the expression of two well known STAT3 downstream target genes implicated in anti-apoptotic (Bcl-xL) and proliferative (Cyclin D1) cancer cell activities after treatment with mc-1Stat3 or dc-Stat3. As shown in Figures 4E and 4F, western blot analysis indicates that expression of Bcl-xL and cyclin D1 is decreased significantly by about 50% after MDA-MB-231 cell treatment with mc-1Stat3 and dc-Stat3 at a concentration near IC20 values for inhibition of cell proliferation (25 nM and 10 nM for dc-Stat3 and mc-1Stat3, respectively); STAT3 target gene expression was statistically dependent on dose treatment for both ODNs ($p < 0.001$). We also studied the expression of STAT3 and p-STAT3 (Y705) under the same conditions of treatment, showing no statistical downregulation of protein expression, which indicates that the decoy activity of both ODNs does not inhibit the expression of total and phosphorylated STAT3. Our data strongly

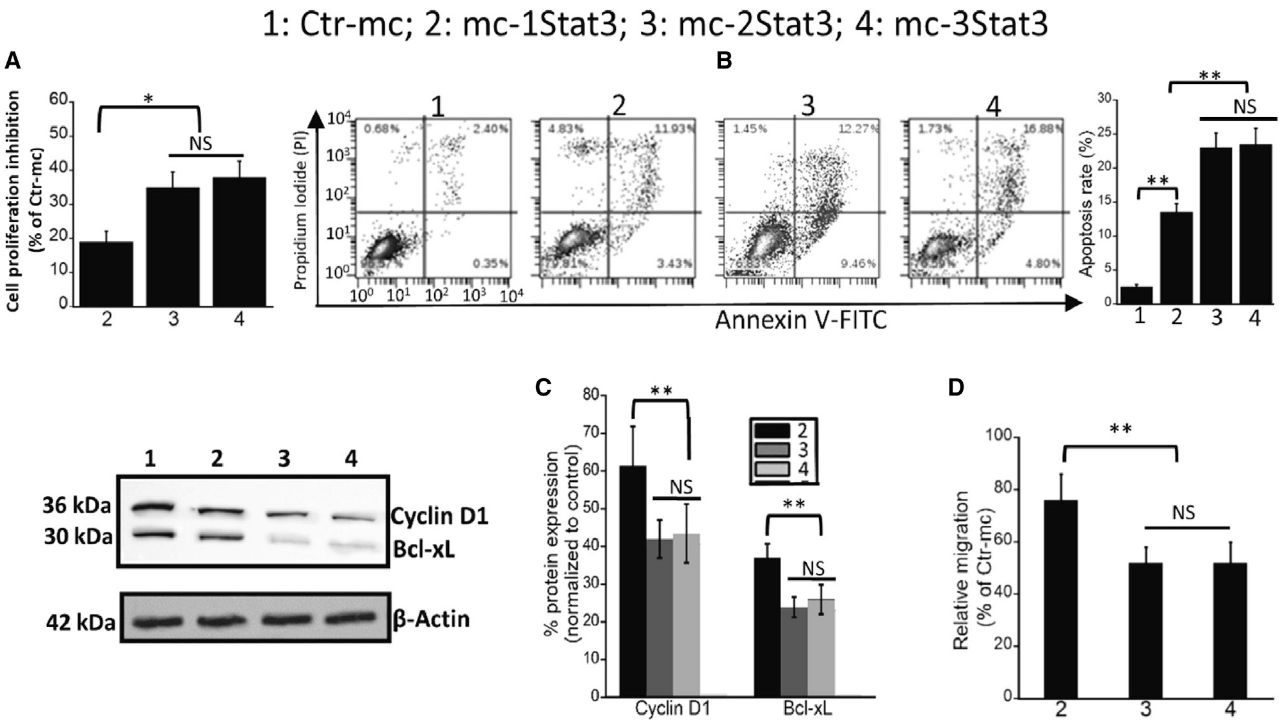

Figure 5. Differential cellular activity of MC as a function of STAT3 binding site number in MDA-MB-231 cells

(A) Comparative proliferation evaluation of MDA-MB-231 cells treated with mc-1Stat3, mc-2Stat3, and mc-3Stat3 (10 nM); cell proliferation was determined 48 h after using the alamarBlue assay. (B) Detection of apoptotic MDA-MB-231 cells by flow cytometry analysis using Annexin V-FITC and propidium iodide. MDA-MB-231 cells were treated with 10 nM of mc-1Stat3, mc-2Stat3, mc-3Stat3, and Ctr-mc; 48 h later, the level of apoptosis was evaluated using the Annexin V/PI dual labeling technique, as determined by flow cytometry. Quantitative results of apoptosis assays are shown on the right as histograms. (C) Expression levels of Bcl-xL and Cyclin D1 after MDA-MB-231 cell treatment with mc-1Stat3, mc-2Stat3, and mc-3Stat3. Shown is a western blot assay of MDA-MB-231 cells treated with MCs bearing one to three STAT3 binding sites and with Ctr-mc at 10 nM concentration. Cell lysates were immunoblotted for the indicated proteins. β-Actin was used as a loading control. (D) Migration inhibition of MDA-MB-231 cells induced by STAT3 MCs. MDA-MB-231 cells were first treated with 10 nM concentration of MCs, and 24 h after treatment, migration was determined using a Transwell migration assay. After 6 h, the migratory cells were counted, and the quantification is shown as a histogram analysis. Results presented are representative of three independent experiments performed in triplicates. All data are expressed as the means ± SD of three independent experiments (*p < 0.05; **p < 0.01; ***p < 0.001).

suggest that the STAT3 target gene expression decrease correlates with STAT3 targeting through the decoy activity of both types of ODNs, with mc-1Stat3 significantly more potent than dc-Stat3. The same conclusions can be drawn from the results obtained when using 4T1 cells, as presented in Figure S8.

MCs carrying two and three STAT3 binding sites exhibit increased activity in TNBC cells

With the aim of enhancing the titration capacity of STAT3 MC, we designed two new 95-bp MC containing two (mc-2Stat3) and three (mc-3Stat3) STAT3 binding sites and analyzed by retardation assay[22] their capacity to bind one and two more STAT3 proteins per MC compared with mc-1Stat3. Following incubation of each MC with recombinant p-STAT3 (Y705), a retarded band is formed for each STAT3 MC, whose mobility is decreasing as a function of the binding site number, indicating binding of one, two, and three STAT3 proteins to mc-1Stat3, mc-2Stat3, and mc-3Stat3, respectively (Figure S9). We next determined whether these new STAT3 MCs could have improved antiproliferative activity compared with mc-1Stat3 in MDA-MB-231 cells. As shown in Figure 4A and as confirmed by dose-response analysis of the three STAT3 MCs, a higher antiproliferative activity was elicited by treatment with mc-2Stat3 and mc-3Stat3 with an IC20 of about 3.5 nM compared with 11 nM for mc-1Stat3 ($p < 0.05$) (Figure S7). The increase in proliferation inhibition of MDA-MB-231 cells induced by mc-3Stat3 compared with mc-2Stat3 was not statistically different. Then we wanted to examine the relationship between the number of STAT3 binding sites and several cellular effects. First, we compared the rate of cell apoptosis induced by the three STAT3 MCs. The rate of MDA-MB-231 apoptosis was increased significantly with mc-2Stat3 and mc-3Stat3 compared with mc-1Stat3 treatment ($p < 0.001$), with an apoptosis rate of nearly 15% with mc-1Stat3 and 25% with mc-2Stat3 and mc-3Stat3 (Figure 5B). The presence of one additive binding site beyond two binding sites per MC did not trigger more apoptosis effects. We determined the decrease in Bcl-xL and Cyclin D1 expression induced by the three STAT3 MCs. As shown in Figure 5C, the decrease in Cyclin D1 expression was significantly higher with mc-2Stat3 treatment (60%) compared with mc-1Stat3 treatment (40%)

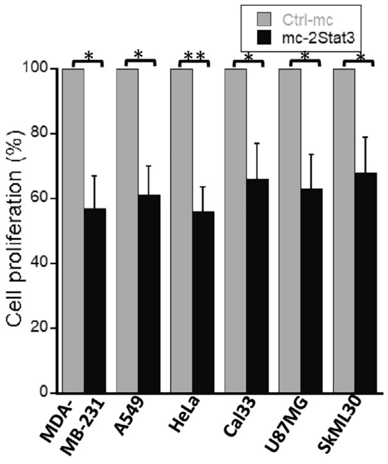

Figure 6. Comparative anti-proliferative effect of mc-2Stat3 on different human cancer cell lines

Various human cancer cell lines were treated with mc-2Stat3 or Ctrl-mc at 10 nM concentration for 48 h, and an alamarBlue assay was used for survival determination. The human cancer lines from various solid tumors were as follows: MDA-MB-231 (TNBC), A549 (lung carcinoma), Cal33 (tongue carcinoma), U87MG (brain glioblastoma), SkML30 (cutaneous melanoma), and HeLa (cervical adenocarcinoma). The experiment was performed in triplicates, and data are mean ± S.D. Statistical significance was determined by unpaired, two-tailed Student's t test for all analysis and by ANOVA analysis for difference between various types of treated cancer cells (*p < 0.05; **p < 0.01; ***p < 0.001).

($p < 0.001$) in MDA-MB-231 cells. The same trend was observed for Bcl-xL expression downregulation. The reduction induced by mc-2Stat3 treatment was 25% and 38% with mc-1Stat3 treatment, whereas the decrease induced by mc-2Stat3 and mc-3Stat3 was not significantly different.

It is well known that STAT3 up regulates several genes that are directly linked to metastasis.[30] To determine whether STAT3 inhibition by decoy MCs could impede MDA-MB-231 cell migration, we evaluated the impact induced by MC carrying one or several STAT3 binding sites by using a Transwell cell migration assay (Figure 5D). The migration of cells treated with mc-2Stat3 or mc-3Stat3 was significantly slower compared with mc-1Stat3 treatment (1.6-fold lower, $p < 0.001$).Our results obtained with MDA-MB-231 cells and 4T1 cells (Figure S10) allowed us to conclude that increasing the number of STAT3 binding sites per MC for dual STAT3 targeting is an efficient strategy to boost decoy MC anticancer activity in TNBC.

MCs carrying two STAT3 binding sites show antiproliferative activity in various cancer cell lines

STAT3 has been shown previously to be persistently activated in several human cancer cell lines.[31,32]. To determine whether STAT3 MC exhibits antiproliferative activity in various human cancer cell lines, we next treated seven different cancer cell lines, including MDA-MB-231 cells, with mc-2Stat3 at a fixed concentration of 10 nM. As shown in Figure 6, all cancer cell lines were statistically equally sensitive to treatment as MDA-MB-231, suggesting that the MC-based STAT3 decoy strategy could be applied not only to TNBC but also to other human tumor types.

STAT3 MC activity on primary tumor growth and metastasis of triple-negative 4T1 mammary carcinoma tumors in mice

Our *in vitro* studies showed that mc-1Stat3 possesses enhanced biological activity with TNBC cells compared with linear dc-Stat3 decoys and that potentiation of STAT3 MC activity can be gained by increasing the number of STAT3 binding sites per MC. To translate these results from *in vitro* to *in vivo* studies, we studied the anticancer effect of mc-1Stat3 treatment in the mammary carcinoma 4T1 model. This murine cancer model shares several characteristics with human triple-negative breast tumors, and the 4T1 syngeneic model is widely used to study spontaneous metastasis of cancer,[33] its major metastatic site being located in lungs.[34] 4T1 cells were implanted orthotopically into mammary fat pads, and the tumor was allowed to grow to palpable size to have the treatment regimen close enough to what exists in clinical treatment situations. We intratumorally (i.t.) administered mc-1Stat3 encapsulated in our homemade lipopolyplex nanoparticle prepared as described previously.[35] The mc-1Stat3 formulation was administered at 250 μg/kg three times per week for 2 weeks into orthotopic tumor-bearing mice, and the growth of the primary tumor was followed in living mice by representative 3D reconstructions of tumor volumes after echography imaging (Figures S11A and S11B). As shown in Figure 7A, treatment with mc-1Stat3 induced a significant reduction in tumor growth compared with Ctrl-mc or vehicle control ($p < 0.001$). mc-1Stat3-treated tumor-bearing mice show a significantly prolonged inhibition effect of tumor growth after treatment arrest from days 21–26, whereas, in the same time period, rapid tumor growth occurred with dc-Stat3. To determine the antimetastatic effect of these different treatments, lungs were collected from the mice, H&E staining was performed on paraffin-embedded lung sections, and the lung metastatic area was quantified (Figure S11C). Figure 7B shows that mc-1Stat3 treatment induced a potent 72% decrease in lung metastasis surface, which is significantly more important than the 45% reduction induced by dc-Stat3 ($p < 0.05$). To determine the effect of decoy treatment on the expression level of STAT3 target genes in 4T1 tumors, the expression of Cyclin D1 and Bcl-xL was determined 48 h after a single i.t. injection of mc-1Stat3 and dc-Stat3. As shown in Figure 7C, the decrease in Cyclin D1 protein expression was significantly higher after mc-1Stat3 treatment compared with dc-Stat3 treatment (50% versus 35%, $p < 0.001$). The same differential effect was also observed for Bcl-xL expression downregulation after tumor treatment by mc-1Stat3 or dc-Stat3. These data strongly suggest that the *in vivo* decoy activity of both types of ODNs inhibits STAT3 activity. Overall, we observed a reduction in tumor growth and lung metastasis surface induced by mc-1Stat3 treatment, which is more effective compared with dc-Stat3. Our data show, for the first time, that mc-1Stat3 is endowed with anticancer activity with a higher effectiveness compared with dc-Stat3.

We next determined whether the presence of several STAT3 binding sites per MC could promote anticancer activity in 4T1 tumor

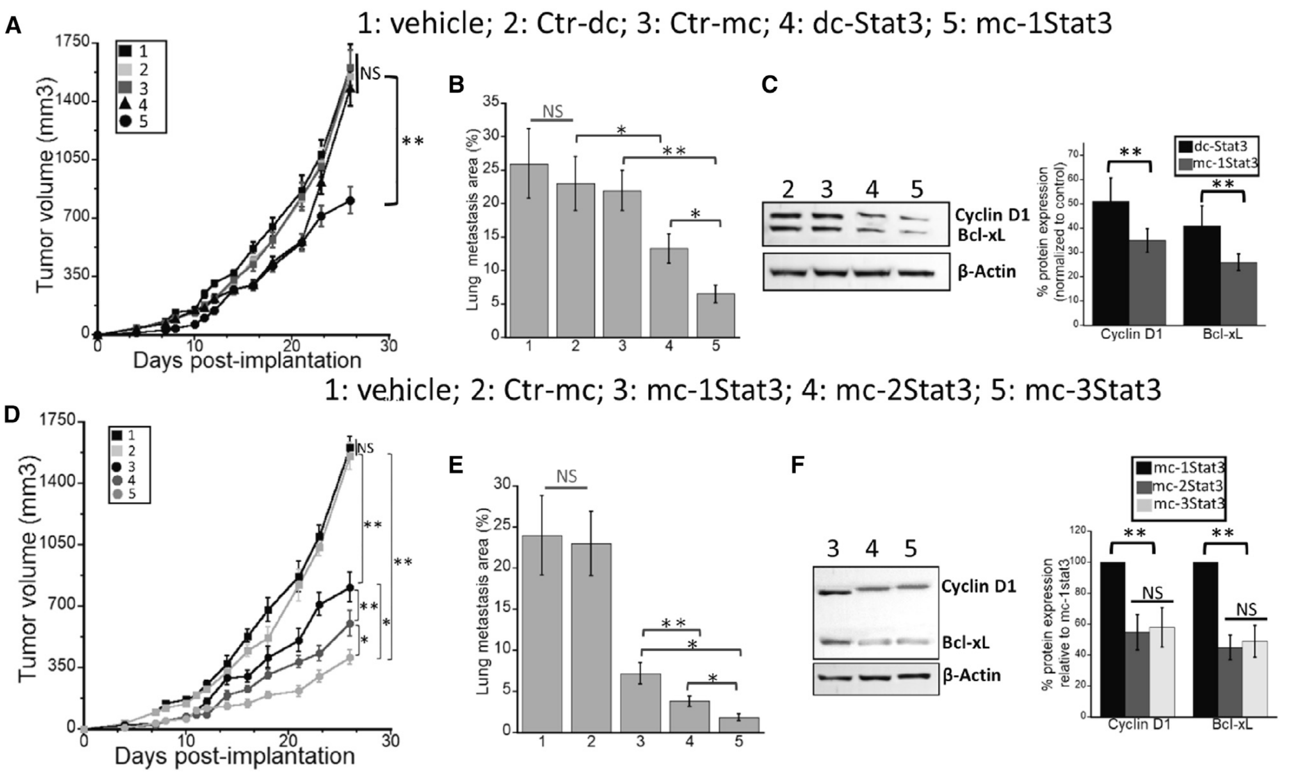

Figure 7. Decoy MCs directed against STAT3 reduce primary tumor growth and metastasis in a murine model of TNBC metastatic breast cancer

4T1 primary tumors were treated by linear and MC decoys as indicated, and primary tumor growth, lung metastasis surface, and expression of the STAT3 target genes Bcl-xL and Cyclin D1 were analyzed. (A and D) Growth curves of untreated and treated 4T1 primary tumors. (B and E) Quantification of lung metastasis surface per lung area 40 days after implantation. (C and F) Western blot from lysates of primary tumors treated with mc-1Stat3, mc-2Stat3, mc-3Stat3, and Ctr-mc; the quantification bar chart analysis is shown on the right. Western blot quantification was carried out relative to mc-1Stat3 in (F) . 3D echography imaging was used to determine the size of 4T1 primary tumors grown within the mammary fat pad as a function of time after i.t. injection of formulated decoy ODNs of interest (i.t. injection on days 8, 10, 12, 14, 16, and 18 of 5 μg of formulated ODN per injection per animal). Data are presented as means with SEM (n = 6 mice/group), and individual treatments were repeated twice. Mice were euthanized 40 days after implantation, and metastasis area/lung area was quantified within each group after H&E staining of lung sections. Western blotting of tumor lysates was carried out 48 h after i.t. injection. Right panel: western blot quantification. A statistically significant difference between treatment groups was found based on ANOVA (*$p < 0.05$; **$p < 0.01$; ***$p < 0.001$).

bearing mice. Figure 7D shows that mc-2Stat3 and mc-3Stat3 treatment enabled a significant increase in tumor growth inhibition compared with mc-1Stat3 ($p < 0.001$ and $p < 0.05$, respectively). The tumor reduction induced by mc-3Stat3 was 2-fold more significant than that resulting from mc-1Stat3 treatment, yielding an overall significant reduction of nearly 80% compared with Ctr-mc-treated groups. As observed in Figure 7E, lung metastasis surface was also significantly more reduced with mc-2Stat3 and mc-3Stat3 treatment compared with mc-1Stat3 ($p < 0.001$), with a total reduction of nearly 75% and 90%, respectively. The mc-2Stat3 and mc-3Stat3 treatments were associated with a significantly more decreased expression of the STAT3 downstream genes cyclin D1 and Bcl-xL compared with mc-1Stat3 ($p < 0.001$). However, no statistical difference was found for either protein expression between treatment with mc-2Stat3 and mc-3Stat3. These results underlie the interest of our decoy strategy based on a single DNA MC carrying several STAT3 binding sites to potentiate antitumor efficacy.

DISCUSSION

In this work, we provide the first demonstration of the therapeutic potential of short DNA MCs as a new type of decoy for TF inhibition. We chose to target STAT3 because there is no direct STAT3 inhibitor available in clinical practice, although this TF is one of the most attractive target for cancer therapy. We also decided to investigate the anticancer effect of the STAT3 decoy strategy in TNBC because a novel molecular targeting approach is urgently needed, and the decoy strategy as a single treatment has not yet been explored for this type of cancer, to the best of our knowledge.

We first assessed that STAT3 MCs delivered into TNBC cells demonstrates molecular properties shared by the parent phosphorothioated STAT3 linear decoy dc-Stat3, whose molecular mechanism and anticancer activity have been studied in pre-clinical and clinical fields.[10] Decoy therapeutic effectiveness depends on several ODN properties, including its cellular uptake and biostability, its binding affinity and specificity for the target protein, and its capacity to downregulate

STAT3 target gene expression. Our data indicate that formulated mc-1Stat3 and linear dc-Stat3 ODNs bearing one identical STAT3 binding sequence were delivered equally into TNBC cancer cells, with similar sublocalization in the cytosol and nucleus (Figures 2A and S3). Both types of ODNs are mostly localized in the cytoplasm, in line with the MC decoy based-action being associated with trapping of cytosolic p-STAT3, as reported in previous data on the decoy mechanism of action.[19,36,37] We found that mc-1Stat3 treatment was more efficient than the linear decoy dc-Stat3 in reducing the viability of MDA-MB-231 and 4T1 TNBC cells and increasing apoptosis with a slowing of the cell cycle. mc-1Stat3 exhibited enhanced cellular stability compared with its linear counterpart dc-Stat3, indicating that STAT3 MCs containing a natural phosphodiester backbone are more stable compared with phosphorothioated linear ODNs (Figure 2B). These data allow us to conclude that the structure of MCs devoid of free ends contributes to enhanced ODN biostability and increased biological activity. This conclusion is reinforced by the fact that the time-dependent proliferation inhibition induced by mc-1Stat3 treatment is longer compared with the linear decoy dc-Stat3 and that the phosphodiester linear STAT3 decoy is very inefficient in reducing cell viability (Figures 3A and 3B). The reduced antiproliferative effect induced by dc-Stat3 compared with mc-1Stat3 could not be attributed to the presence of phosphorothioate modification because it does not have any effect on STAT3 binding activity when present in the terminal part of ODNs.[10] Our data show that mc-1Stat3 can specifically bind to the cellular form of phosphorylated STAT3 (Y705) because no substantial binding occurs with control MCs devoid of any specific binding site (Figure 2C). In line with this finding, the control MCs were also unable to induce cell proliferation reduction despite the fact that their cellular uptake was similar to that of mc-1Stat3. Mc-1Stat3 treatment was also shown to specifically decrease Cyclin D1 and Bcl-xL expression, two well-known STAT3-dependent transcription target genes (Figure 4F). These first results show that STAT3 MC acts as a specific STAT3 decoy and allow us to assume that the circular shape of MCs does not trigger nonspecific effects that could induce nonspecific cellular responses in the range of nucleic acid concentration used here. We administered i.t. the formulated ODN of interest to investigate the antitumoral potential of the MC-based strategy. This type of administration is independent of delivery route and systemic side effects and was performed successfully in several pre-clinical studies to investigate the antitumoral potential of novel oligonucleotide-based strategies.[38–41] I.t. injection was shown to deliver elevated therapeutic oligonucleotide concentrations into the tumor without detectable accumulation in mouse organs.[41,42] In line with these results, formulated mc-1Stat3 labeled with fluorescent dye was found to be localized in the tumor after i.t. injection into 4T1 tumor-bearing mice (data not shown). Mc-1Stat3 treatment is more efficient in decreasing tumor growth and lung metastasis area compared with dc-Stat3 treatment (Figures 7A and 7B), which is consistent with the higher biostability of mc-1Stat3 compared with dc-Stat3 observed in TNBC cells treated *in vitro*. We observed that downregulation of the STAT3 target genes Bcl-xL and Cyclin D1 was correlated with mc-1Stat3 activity after treatment of 4T1 cancer cells *in vitro* and also of 4T1 cell-bearing mice. This correlation strongly suggests that the growth of 4T1 cancer cells in the tumor is inhibited after treatment with mc-1Stat3 through STAT3 targeting. In line with the likelihood that STAT3 inhibition is induced by mc-1Stat3 in 4T1 cell-bearing mice, a reduction of primary tumor growth and a significant decrease in lung metastasis have been reported when STAT3 was knocked down in 4T1 cell-bearing mice.[43,44] However, given the importance of STAT3 activity in regulating several types of non-cancer cells present in the tumor microenvironment, such as immune cells[45] and tumor-associated fibroblasts,[46] it is likely that mc-1Stat3 could directly and/or indirectly inhibit STAT3 activity in different types of non-cancer cells. Metastasis spread has been shown to be potentiated in 4T1 cell-bearing mice by tumor myeloid-derived tumor cells implicated in tumor immunosuppression.[43] Therefore, inhibition of tumor myeloid cells through STAT3 targeting by mc-1Stat3 could explain why the relevant regression of lung metastasis surface induced by mc-1Stat3 treatment contrasts with the weak reduction of 4T1 TNBC cancer cell migration we observed *in vitro*. Our findings indicate *in vivo* anticancer efficacy of mc-1Stat3 treatment over the parent dc-Stat3 decoy without induction of nonspecific effects, as shown upon treatment with the control MCs, suggesting that MC anticancer activity could be further enhanced through dose escalation.

An important aspect of using MCs as decoys relates to their size and shape, which enables insertion of several TF binding sequences per MC to develop a TF multitargeting approach,[22] In the present study, we designed mc-2Stat3 and mc-3Stat3 to potentiate the decoy activity of mc-1Stat3. Compared with mc-1Stat3, mc-2Stat3 was found to be more effective in inhibiting proliferation and inducing apoptosis in MDA-MB-231 and 4T1 cells. Increased activity *in vitro* with mc-2Stat3 compared with mc-1Stat3 treatment was also observed *in vivo*; primary tumor volume and lung metastasis surface were reduced by nearly 30% and 50%, respectively (Figures 7D and 7E). Compared with the control group treated with Ctr-mc, mc-3Stat3 treatment induced the most effective anticancer activity, with 80% and 90% reduction of tumor growth and metastasis surface, respectively. Therefore, the STAT3 multitargeting strategy using mc-3Stat3 treatment significantly improved mc-1Stat3 antitumoral activity by nearly 50% and 75% in reducing tumor growth and lung metastasis, respectively. Our *in vivo* data indicate that mc-3Stat3 treatment exhibited significantly more anticancer activity compared with mc-2Stat3, suggesting an increase in STAT3 titration through the presence of a third binding site. However, this gain of activity was found to be not statistically significant after *in vitro* treatment, although mc-3Stat3 was able to bind three STAT3 proteins, as shown by a retardation assay (Figure S9). A plausible explanation is that the difference between *in vivo* and *in vitro* data may arise from an additive treatment effect obtained *in vivo* through time-dependent repeated treatment with mc-3Stat3 versus a single treatment carried out *in vitro*. This is in agreement with the fact that repetitive doses of STAT3 decoys have been shown previously to promote *in vivo* treatment.[37] However, this explanation does not rule out that the third sequestering binding site in mc-3Stat3 exhibited lower affinity toward cellular p-STAT3 (Y705) *in vitro*. It is likely that the decrease in available cellular

p-STAT3 (Y705) through titration activity of two binding sites could reduce STAT3 TF binding to genomic promoters and to the third binding site of mc-3Stat3. Such a view is reinforced by recent genomic-wide binding studies indicating that TF is not present in excess into cell but as limited pool of active DNA binding TF.[47] Our *in vitro* and *in vivo* data show strong enhancement of anticancer activity induced by the STAT3 multitargeting capacity of a single MC containing two and three STAT3 binding sites.

This work shows, for the first time, that the unique structure of small DNA MCs enables design of a new generation of antitumor decoy ODNs without chemical modifications and free ends. Compared with the first-generation STAT3 decoy, MCs exhibit higher anticancer activity because of its greater resistance to cellular nucleases. There is also a possibility of bearing several TF binding sites on a single MC, leading to multitargeting capacity. Such a multitargeting strategy could be further applied to inhibit different types of TFs, such as STAT3 and NF-κB, by a single MC; these TFs are known to cooperate in inducing TNBC progression.[48] Several important parameters, including the mode of delivery, the nature of the formulations,[49] and the selective targeting of cells in the tumor microenvironement,[50] control the efficacy of small nucleic acids. Intravenous delivery needs to be developed especially in the field of cancer therapy. Toward this goal, the size and production method of MCs offer the opportunity to use them as a platform for versatile incorporation of TF binding sites for decoy activity together with site-specific modification, such as cholesteryl residues[22] and tumor-targeting ligands, for optimal tumor delivery. Increasing selective delivery of STAT3 MCs to the tumor as much as possible is of great importance for targeting survival signals and immune checkpoints directly in TNBC cells and indirectly in cancer-associated immunosuppressive cells.

MATERIALS AND METHODS

Cell culture and reagent

All cell lines used in this work were from the ATCC, except Cal33, which was kindly provided by Prof. G. Milano (Nice, France). Cell authentication was carried out by genetics characterization (Eurofins, Ebersberg, Germany). All cell lines were cultured in medium as recommended by the ATCC. The MDA-MB-231 and 4T1 cell lines were cultured in DMEM high glucose and RPMI medium, respectively, and both media were purchased from Gibco (Thermo Fisher Scientific, Waltham, MA). All cell culture media contained 10% fetal bovine serum and 1% penicillin/streptomycin. Cells were grown at 37°C in a humidified atmosphere containing 5% CO_2. Cells were mycoplasma free as determined by the mycoAlert Mycoplasma detection kit (Lonza).

Linear and MC ODNs

Single-stranded ODNs from Eurogentec were synthesized with or without chemical modifications, as indicated in the text. 15-mer DNA duplexes were formed by hybridization of the complementary strands added in equimolar amounts at a final concentration of 20 μM, followed by slow cooling from 80°C to 15°C in buffer containing 25 mM NaCl and 10 mM Tris-HCl (pH 7.5). For the ODN sequence composition incorporated into the MCs designed for STAT3-dependent biochemical and biological studies, we used the Jaspar database (http://jaspar.binf.ku.dk) to select MC sequences with the highest and lowest score for specific Stat3 binding and nonspecific sequences, including the design of control MCs, respectively (Figure S5). Proteinase K, T4 DNA ligase, T4 polynucleotide kinase, and Exonuclease III were purchased from Thermo Fisher Scientific, and T5 exonuclease and the low-molecular-weight DNA ladder were from New England Biolabs.

Preparation of 95-bp relaxed DNA MC was carried out as reported previously.[21] Briefly, nicked DNA templates was performed by mixing equimolar quantities of complementary single-stranded oligonucleotides in buffer containing 10 mM Tris-HCl and 25 mM NaCl (pH 7.5) at a concentration of 40 μM; hybridization was carried out by slowly cooling the oligonucleotide mixture from 80°C to 15°C. A standard circularization reaction was carried out by adding one of the overlapping nicked duplexes shown in the supplemental information at a final concentration of 2 μM in a buffer containing 40 mM Tris-HCl, 10 mM $MgCl_2$, 10 mM DTT, 0.5 mM ATP, and 5% glycerol (pH 7.8). Abf2p was then added to a final concentration of 3 μM, followed by T4 DNA ligase addition (10 units); the reaction mixture was then incubated at 20°C for 1 h, yielding nicked MC intermediates. Next, the reaction mixture was sequentially incubated with proteinase K (0.3 units, 30 min at 55°C), T4 polynucleotide kinase with 1 mM ATP (100 units, 30 min at 37°C), T4 DNA ligase (25 units, 1 h at 20°C), and exonuclease T5 (200 units, 30 min at 37°C) to obtain covalently closed MCs after carrying out a final incubation with proteinase K. The MCs were then precipitated by addition of $AcONH_4$ to a final concentration of 2.5 M, followed by addition of 2 volumes of cold ethanol. After centrifugation, the pellet was washed by addition of 70% ethanol and then resuspended in buffer containing 10 mM Tris-HCl and 1 mM EDTA (pH 7.5); the optical density of the dsMC sample was measured to determine its concentration and to ensure that the optical density 260/280 ($OD_{260/280}$) was greater than 1.8, corresponding to high-quality DNA. For confocal microscopy and flow cytometry analysis, FITC-labeled ODNs were used. For pull-down assays, biotinylated ODNs were used.

ODN formulation and *in vitro* uptake

MDA-MB-231 or 4T1 cells were plated on a 24-well plate at 500 μL/well at density of 12×10^4 cells/mL in the indicated growth medium, and cells were grown for 24 h until the time of transfection. Linear or circular DNA ODNs were mixed with Lipofectamine 3000 (Thermo Fisher Scientific), and the complex was prepared according to the manufacturer's protocol. Briefly, 1 μg of DNA decoy was complexed with 2 μL of Lipofectamine 3000 in Opti-MEM reduced serum medium, and after 15 min of incubation at room temperature, complexes were added to plated cells. The final ODN concentration and incubation time at 37°C are indicated in the text. All transfection assays were carried out in triplicate simultaneously.

Intracellular uptake of linear and MC DNA decoys was studied by microscopy using a Carl Zeiss Axiovert 200M inverted microscope

(equipped with a Plan-Apochromat 63× objective, 1.4 na) coupled with an LSM 510 scanning confocal head (Carl Zeiss). MDA-MB-231 or 4T1 cells were first seeded in 4-well Lab-Tek chambered cover glass (Nunc), and 24 h later, fluorescein-labeled linear and circular DNA decoys at a concentration of 15 nM were transfected in the presence of Lipofectamine 3000. 6 h after transfection, cells were fixed in 3% paraformaldehyde (PFA) in PBS solution at room temperature for 10 min, and excess PFA was removed with 3 washes of 5 min in PBS. For nuclear counterstaining, cells were incubated with 300 nM DAPI (Thermo Fisher Scientific) stain solution for 5 min at room temperature. Fluorescence microscopy imaging was then carried out using the appropriate filters as indicated.

Flow cytometry, cell viability, and migration assay

Flow cytometry analysis was used to determine apoptosis of tumor cells and cell cycle distribution after 48 h of treatment with the different Stat3 decoy ODNs and their respective control forms as indicated. The percentage of apoptotic cells was determined using an Annexin V-FITC/PI apoptosis assay (Abcam). Cells were harvested, washed with PBS, and stained with Annexin V/PI. Apoptosis of TNBC cells was detected by determining the relative amount of Annexin V-FITC-positive cells alone (early apoptosis) and of Annexin V-FITC- and PI-positive cells (late apoptosis). Experiments were done twice in triplicate.

For cell cycle analysis, treated cells were harvested, resuspended in PBS, and fixed in 70% ethanol. After washing in PBS, cells were resuspended in PBS and stained with a solution containing 100 μg/mL RNase A, 100 μg/mL propidium iodide, and 0.1% Triton X-100. Cells were incubated for 5 min in the dark at 4°C and transferred into a flow cytometry tube. For both types of cell analyses, a cytometer (FACSort; Becton Dickinson, Franklin Lakes, NJ, USA) was used, and cell fluorescence intensity was measured (λex = 488 nm, λem = 530 ± 30 nm for FITC fluorescence; λex = 488 nm, λem = 617 nm for propidium iodide). Cell cycle distribution was determined by using ModFit LT software.

Cell viability was determined using the alamarBlue assay. Briefly, cells were seeded in 24-well plates and transfected with a single dose or increasing concentrations of DNA decoy ODNs, followed by the alamarBlue assay. After 48 h or at selected time points as specified in the text, 50 μL of alamarBlue solution was added to 500 μL of culture medium and incubated for 1 h at 37°C. Fluorescence (Ex 530 nm, emission, 590 nm) was measured on a Victor microplate reader (PerkinElmer). For each experiment, control experiments were made consisting untreated cells and cells treated with nonspecific control oligonucleotides as a negative control. The data were normalized to their respective control oligonucleotides. The percentage of cell proliferation inhibition was deduced from the percentage of cell proliferation treated with the indicated specific decoy at a time point and from the percentage of cell proliferation treated with the corresponding nonspecific oligonucleotide at the same time point to yield the percentage of cells that have not contributed to proliferation. In all experiments, the toxic effect induced by nonspecific oligonucleotide was less than 20% compared with untreated cells. Under our assay conditions, the number of cells was linearly proportional to the fluorescence signal induced by the alamarBlue reagent.

In vitro Transwell migration of MDA-MB-231 cells was performed using a Transwell unit (8-μm pore size membrane filters; Corning, MA, USA). The membranes were washed thoroughly in PBS and dried immediately before use. Cells were transfected for 48 h as described above. Trypsinized cells (3×10^4) were then suspended in 500 μL of serum-free medium and seeded onto the membranes of the upper chambers, which had been inserted into the wells of 24-well plates containing 10% fetal bovine serum (FBS)-supplemented medium. After 12 h, unmigrated cells in the upper chambers of each insert were removed by wiping with a damp cotton swab. The cells that migrated to the lower surface of the membrane were fixed with methanol for 10 min and stained with DAPI (4',6-diamidino-2-phenylindole, Sigma-Aldrich) before imaging of the membrane with a Video Observer Z1 videomicroscope (Carl Zeiss). All experiments were done in triplicate, and a minimum of five fields per membrane were counted using ImageJ software.

ODN stability assay with whole-cell lysate

The biostability of ODNs in MDA-MB-231 cells was determined with whole-cell lysate prepared by using the Native Lysis Buffer Kit (Abcam) according to the supplier's recommendations. 365 ng of DNA was incubated in 90 μL of whole-cell extract from MDA-MB-231 cells at 37°C. Aliquots were taken as a function of time and immediately deproteinized twice by adding a mixture of phenol-chloroform-isoamyl alcohol (25:24:1) (Invitrogen). After centrifugation, an aliquot of the supernatant was loaded on polyacrylamide gel (5% and 15% denaturing polyacrylamide gel for MCs and linear DNA duplexes, respectively). After electrophoresis, the gel was stained with SYBR Green (Invitrogen) and imaged with a Typhoon Trio (GE Healthcare), and quantification of the gel bands was performed by ImageQuant software v.5.1. The band intensity corresponding to the starting nucleic acid of interest was reported as a function of incubation time. The data points were fitted to a single exponential. The rate constants of nucleic acid disappearance were used to deduce the half-time of nucleic acid stability in cell extract.

Western blotting and ODN pull-down

MDA-MB-231 or 4T1 cells were seeded at 6×10^4 cells in 24-well plates, and 24 h later, they were transfected with the formulated ODN of interest at the indicated concentration with a final volume of 500 μL in Opti-MEM for 4 h, and then complete medium was added. 48 h later, cell pellets were resuspended in 100 μL RIPA buffer (Thermo Fisher) containing protease inhibitors and incubated on ice for 30 min. Cell lysates were extracted and protein concentrations quantified using the BCA Assay Kit (Thermo Fisher Scientific). Whole-cell lysates (40 μg protein/sample) were mixed with an equal volume of Laemmli buffer (Bio-Rad, Hercules, CA) supplemented with 10% β-mercaptoethanol (Sigma) and electrophoresed on 8% SDS-polyacrylamide gel assembled in a reservoir containing 1× Tris/glycine buffer (Bio-Rad). The SDS-PAGE gel was run for 1.5 h

at 150 V and transferred onto a Trans-Blot polyvinylidene difluoride (PVDF) membrane (Bio-Rad) with a TransBlot turbo (Bio-Rad). The membrane was then placed in blocking milk buffer containing 5% nonfat dry milk and 0.1% Tween 20 in PBS (TBST) for 20 min, followed by shaking for 1 h at room temperature. After blocking, the membranes were incubated with a diluted primary antibody purchased from Cell Signaling Technology (p-STAT3 [Y705], 1:1,000, MW 79.9 kDa; catalog number 9145S; STAT3, 1:2,000, MW 79.9 kDa, catalog number 12640; Bcl-xL, 1:2,000, 30 kDa, catalog number 2762; Cyclin D1, 1:2,000, MW 36 kDa, catalog number 2922; β-actin, 1:5,000, MW 45 kDa, catalog number 3700 as loading control) overnight at 4°C. The membrane was washed three times in TBST (10 min/wash), followed by incubation with anti-rabbit secondary antibody (1:4,000) for 1 h at room temperature. The membrane was again washed three times for 10 min. Then the membrane was incubated with Clarity western enhanced chemiluminescence (ECL) substrate (Bio-Rad) for the chemiluminescence assay according to the manufacturer's protocol. Blot imaging was performed with the Pxi imaging system (Syngene), and the relative level of protein expression was quantified with ImageJ software using β-actin as a loading control.

For pull-down assays, preparation of nuclear protein fractions from MDA-MB-231 cells was performed using NE-PER nuclear and cytoplasmic extraction reagents from Pierce (Thermo Fisher Scientific) following the manufacturer's instructions. 200 ng of STAT3 MC ODNs containing a biotin residue (biotinylated mc-1Stat3) was incubated with 25 μg of nuclear extract in buffer containing 150 mM NaCl, 0.1 mM EDTA, 10 mM NaF, 10 μM Na_2MoO_4, 1 mM $NaVO_3$, 1 mM DTT, 15% glycerol, 10 mM HEPES (pH 7) and protease inhibitors at 4°C for 20 min in the presence or absence of nonbiotinylated ODNs as specific or nonspecific competitors. The complexes were captured by incubation with 40 μL of streptavidin magnetic beads (Ademtech, France) for 10 min at 4°C. After extensive washes with binding buffer, complexes were separated on SDS-polyacrylamide gel (8%), and subjected to immunoblotting using anti-p-STAT3 antibody and antibody directed to β-actin as a loading control. Results were analyzed by chemiluminescence as described above for western blotting.

In vivo antitumor activity

Animal housing was provided by our animal facility (accreditation number D-45-234-12, Chantal Pichon) according to the guidelines of the French Ministry of Agriculture for experiments with laboratory animals (approval number 13749). Six-week-old female BALB/c mice (Charles River Laboratories, France) were allowed to acclimatize for 1 week and kept in individual ventilated cages with free access to food and water. Next, mice were inoculated orthotopically into the fourth mammary fat pad with 5×10^5 4T1 cells per mouse after depilation. After 1 week, animals with palpable tumors were randomized into treatment and vehicle control groups (6 mice per group) after 3D tumor size determination by echography and representative 3D reconstruction of tumor volumes (Vevo 2100, Fujifilm VisualSonics). Each mouse was injected i.t. on days 8, 10, 12, 14, 16, and 18 with formulated oligonucleotide as indicated (250 μg/kg/injection). Lipopolyplexes formulations resulting from association of MCs or linear decoy ODNs with a cationic polymer and a mixture of two cationic liposomes were prepared as described previously.[35] Briefly, polyplex-containing decoy ODNs were first obtained by mixing 15 μg of histidinylated linear polyethyleneimine (Polytheragene, Evry, France) with 5 μg of decoy ODNs in a final volume of 20 μL containing 60 mM NaCl, 5% glucose, and 10 mM HEPES (pH 7.4) (decoy/polymer weight ratio 1/3). After 10 s of vortexing, the solution was kept at 20°C for 30 min. Then, 5 μL of Liposome 100 prepared as a liposomes stock solution of 5.4 mM was mixed with the polyplexes (decoy/lipid charge ratio of 1/2) and kept for 15 min at 20°C. 25 μL of this formulation was injected i.t. with a 500 μL insulin syringe. The vehicle control group received the medium used for ODN injection. After 40 days of tumor challenge, mice were sacrificed by cervical dislocation, and the lungs were removed from each mouse and fixed in PFA. To evaluate metastatic spread to the lungs, paraffin embedding, tissue sectioning into 5-μm sections, H&E staining, and mounting of lung tissue sections on microscope slides were carried out by the Histology Laboratory facility of the Anatomopathology Department of the Centre hospitalier régional d'Orléans (France). Imaging of lung tissue sections was carried out with a Video Observer Z1 videomicroscope (Carl Zeiss), and then metastatic dark purple areas were counted using ImageJ software. Western blot analyses were also conducted to detect expression of the STAT3 target genes Bcl-xL and Cyclin D1 in 4T1 primary tumors. Briefly, mice bearing 4T1 primary tumors of 100 ± 20 mm^3 were injected i.t. as described above with various ODNs and their respective control, as indicated. 48 h later, mice were sacrificed, and tumors were resected. Tumor pieces were homogenized with a Precellys homogenizer in RIPA buffer. Western blotting was then carried out as described above.

Statistical analysis

For statistical analysis, data were reported as mean ± standard deviation (SD) or standard error of the mean (SEM). Statistical significance was assessed using a one-way analysis of variance (ANOVA) among three or more groups or Student's t test between two groups using Origin v.9.0 software (Microcal). When ANOVA demonstrated a significant difference among the groups, including control groups, post hoc Tukey's test was performed.

SUPPLEMENTAL INFORMATION

Supplemental information can be found online at https://doi.org/10.1016/j.omtn.2022.06.012.

ACKNOWLEDGMENTS

We are indebted to David Gosset for technical assistance with confocal microscopy analysis and Anthony Delalande for assistance with echographic measurements. We also thank Rudy Clémençon for help with animal experiments. Cal33 was kindly provided by Prof. Gérard Milano (Laboratory of Oncopharmacology, Centre Antoine Lacassagne, Nice, France). We also thank Dr. Patrick Michenet for lung section H&E staining (Anatomopathology Department of the Centre hospitalier régional, Orléans, France). This study was

supported by the Ligue contre le cancer Région Centre et Loiret (to P.M., F.P., and J.-M.M.]. PhD funding (to G.C.) was from the Ministère de l'Enseignement Supérieur et de la Recherche and the University of Orléans.

AUTHOR CONTRIBUTIONS

Conceptualization, P.M., C.P., and J.-M.M.; experimental conception and design, G.C., F.P., and J.-M.M.; writing – original draft, G.C. and J.-M.M.; supervision, P.M., C.P., and J.-M.M.; funding acquisition, P.M., C.P., and J.-M.M. All authors have read and agree to the published version of the manuscript.

DECLARATION OF INTERESTS

The authors declare no competing interests.

REFERENCES

1. Smith, C.I.E., and Zain, R. (2019). Therapeutic oligonucleotides: state of the art. Annu. Rev. Pharmacol. Toxicol. *59*, 605–630.
2. Roberts, T.,C., Langer, R., and Wood, M.,J.,A. (2020). Advances in oligonucleotide drug delivery. Nat. Rev. Drug Discov. *19*, 673–694.
3. Rossi, J.,J., and Rossi, D.,J. (2021). siRNA drugs: here to stay. Mol. Ther. *29*, 431–432.
4. Hecker, M., and Wagner, A.,H. (2017). Transcription factor decoy technology : a therapeutic update. Biochem. Pharmacol. *144*, 29–34.
5. Lau, Y.K., Ramaiyer, M., Johnson, D.E., and Grandis, J.R. (2019). Targeting STAT3 in cancer with nucleoside therapeutics. Cancers *11*, 1681.
6. Lee, T.I., and Young, R.A. (2013). Transcriptional regulation and its misregulation in disease. Cell *152*, 1237–1251.
7. Lambert, M., Jambon, S., Depauw, S., and David-Cordonnier, M.H. (2018). Targeting transcription factors for cancer treatment. Molecules *23*, 1479.
8. Hoel, A.W., and Conte, M.S. (2007). Edifoligide: a transcription factor decoy to modulate smooth muscle cell proliferation in vein bypass. Cardiovasc. Drug Rev. *25*, 221–234.
9. Mamet, J., Harris, S., Klukinov, M., Yeomans, D.C., Donahue, R.R., Taylor, B.K., Eddinger, K., Yaksh, T., and Manning, D.C. (2017). Pharmacology, pharmacokinetics, and metabolism of the DNA-decoy AYX1 for the prevention of acute and chronic post-surgical pain. Mol. Pain *13*, 1–16.
10. Sen, M., Thomas, S.M., Kim, S., Yeh, J.I., Ferris, R.L., Johnson, J.T., Duvvuri, U., Lee, J., Sahu, N., Joyce, S., et al. (2012). First-in-human trial of a STAT3 decoy oligonucleotide in head and neck tumors: implications for cancer therapy. Cancer Discov. *2*, 694–705.
11. Osako, M.K., Tomita, N., Nakagami, H., Kunugiza, Y., Yoshino, M., Yuyama, K., Tomita, T., Yoshikawa, H., Ogihara, T., and Morishita, R. (2007). Increase in nuclease resistance and incorporation of NF-kappaB decoy oligodeoxynucleotides by modification of the 3'-terminus. J. Gene Med. *9*, 812–819.
12. Sasaki, A., and Kinjo, M. (2010). Monitoring intracellular degradation of exogenous DNA using diffusion properties. J. Control Release *143*, 104–111.
13. Eckstein, F. (2014). Phosphorothioates, essential components of therapeutic oligonucleotides. Nucleic Acid Ther. *24*, 374–387.
14. Miyake, T., Aoki, M., Osako, M.K., Shimamura, M., Nakagami, H., and Morishita, R. (2011). Systemic administration of ribbon-type decoy oligodeoxynucleotide against nuclear factor κB and ets prevents abdominal aortic aneurysm in rat model. Mol. Ther. *19*, 181–187.
15. Crinelli, R., Bianchi, M., Gentilini, L., Palma, L., Sørensen, M.D., Bryld, T., Babu, R.B., Arar, K., Wengel, J., and Magnani, M. (2004). Transcription factor decoy oligonucleotides modified with locked nucleic acids: an *in vitro* study to reconcile biostability with binding affinity. Nucleic Acids Res. *32*, 1874–1885.
16. Chu, B.C., and Orgel, L.E. (1992). The stability of different forms of double-stranded decoy DNA in serum and nuclear extracts. Nucleic Acids Res. *20*, 5857–5868.
17. Miyake, T., Miyake, T., Sakaguchi, M., Nankai, H., Nakazawa, T., and Morishita, R. (2018). Prevention of asthma exacerbation in a mouse model by simultaneous inhibition of NF-κB and STAT6 activation using a chimeric decoy strategy. Mol. Ther. Nucleic Acids *10*, 159–169.
18. Mariot, V., Joubert, R., Marsollier, A.C., Hourdé, C., Voit, T., and Dumonceaux, J.A. (2020). Deoxyribonucleic acid decoy trapping DUX4 for the treatment of facioscapulohumeral muscular dystrophy. Mol. Ther. Nucleic Acids *22*, 1191–1199.
19. Zhang, Q., Hossain, D.,M., Duttagupta, P., Moreira, D., Zhao, X., Won, H., Buettner, R., Nechaev, S., Majka, M., Zhang, B., et al. (2016). Serum-resistant CpG-STAT3 decoy for targeting survival and immune checkpoint signaling in acute myeloid leukemia. Blood *127*, 1687–1700.
20. Sen, M., Paul, K., Freilino, M.L., Li, H., Li, C., Johnson, D.E., Wang, L., Eiseman, J., and Grandis, J.R. (2014). Systemic administration of a cyclic STAT3 decoy oligonucleotide inhibits tumor growth without inducing toxicological effects. Mol. Med. *20*, 46–56.
21. Zhao, N., Fogg, J.M., Zechiedrich, L., and Zu, Y. (2011). Transfection of shRNA-encoding Minivector DNA of a few hundred base pairs to regulate gene expression in lymphoma cells. Gene Ther. *18*, 220–224.
22. Thibault, T., Degrouard, J., Baril, P., Pichon, C., Midoux, P., and Malinge, J.M. (2017). Production of DNA minicircles less than 250 base pairs through a novel concentrated DNA circularization assay enabling minicircle design with NF-κB inhibition activity. Nucleic Acids Res. *45*, e26.
23. Yu, H., Lee, H., Herrmann, A., Buettner, R., and Jove, R. (2014). Revisiting STAT3 signalling in cancer: new and unexpected biological functions. Nat. Rev. Cancer *14*, 736–14746.
24. Tošić, I., and Frank, D.A. (2021). STAT3 as a mediator of oncogenic cellular metabolism: pathogenic and therapeutic implications. Neoplasia *23*, 1167–1178.
25. Johnson, D.E., O'Keefe, R.A., and Grandis, J.R. (2018). Targeting the IL-6/JAK/STAT3 signalling axis in cancer. Nat. Rev. Clin. Oncol. *15*, 234–248.
26. Qin, J.J., Yan, L., Zhang, J., and Zhang, W.D. (2019). STAT3 as a potential therapeutic target in triple negative breast cancer: a systematic review. J. Exp. Clin. Cancer Res. *38*, 195.
27. Marotta, L.L., Almendro, V., Marusyk, A., Shipitsin, M., Schemme, J., Walker, S.R., Bloushtain-Qimron, N., Kim, J.J., Choudhury, S.A., Maruyama, R., et al. (2011). The JAK2/STAT3 signaling pathway is required for growth of CD44+CD24- stem cell-like breast cancer cells in human tumors. J. Clin. Invest. *121*, 2723–2735.
28. Ma, J.H., Qi, J., Lin, S.Q., Zhang, C.Y., Liu, F.Y., Xie, W.D., and Li, X. (2019). STAT3 targets *ERR-α* to promote epithelial-mesenchymal transition, migration, and invasion in triple-negative breast cancer cells. Mol. Cancer Res. *17*, 2184–2195.
29. Sirkisoon, S.R., Carpenter, R.L., Rimkus, T., Anderson, A., Harrison, A., Lange, A.M., Jin, G., Watabe, K., and Lo, H.W. (2018). Interaction between STAT3 and GLI1/tGLI1 oncogenic transcription factors promotes the aggressiveness of triple-negative breast cancers and HER2-enriched breast cancer. Oncogene *37*, 2502–2514.
30. Carpenter, R.L., and Lo, H.W. (2014). STAT3 target genes relevant to human cancers. Cancers *6*, 897–925.
31. Bromberg, J. (2002). Stat proteins and oncogenesis. J. Clin. Invest. *109*, 1139–1142.
32. Buettner, R., Mora, L.B., and Jove, R. (2002). Activated STAT signaling in human tumors provides novel molecular targets for therapeutic intervention. Clin. Cancer Res. *8*, 945–954.
33. Arroyo-Crespo, J.J., Armiñán, A., Charbonnier, D., Deladriere, C., Palomino-Schätzlein, M., Lamas-Domingo, R., Forteza, J., Pineda-Lucena, A., and Vicent, M.J. (2019). Characterization of triple-negative breast cancer preclinical models provides functional evidence of metastatic progression. Int. J. Cancer *145*, 2267–2281.
34. Aslakson, C.J., and Miller, F.R. (1992). Selective events in the metastatic process defined by analysis of the sequential dissemination of subpopulations of a mouse mammary tumor. Cancer Res. *52*, 1399–1405.
35. Gonçalves, C., Berchel, M., Malard, V., Cheradame, H., Jaffrès, P.A., Guégan, P., Pichon, C., and Midoux, P. (2014). Lipopolyplexes comprising imidazole/imidazolium lipophosphoramidate, histidinylated polyethyleneimine and siRNA as efficient formulation for siRNA transfection. Int. J. Pharm. *460*, 264–272.
36. Souissi, I., Najjar, I., Ah-Koon, L., Schischmanoff, P.,O., Lesage, D., Le Coquil, S., Roger, C., Dusanter-Fourt, I., Varin-Blank, N., et al. (2011). A STAT3-decoy

oligonucleotide induces cell death in a human colorectal carcinoma cell line by blocking nuclear transfer of STAT3 and STAT3-bound NF-κB. BMC Cell Biol. *12*, 14–32.

37. Njatcha, C., Farooqui, M., Kornberg, A., Johnson, D.E., Grandis, J.R., and Siegfried, J.M. (2018). STAT3 cyclic decoy demonstrates robust antitumor effects in non-small cell lung cancer. Mol. Cancer Ther. *17*, 1917–1926.

38. Zhang, M., Yan, L., and Kim, J.,A. (2015). Modulating mammary tumor growth, metastasis and immunosuppression by siRNA-induced MIF reduction in tumor microenvironment. Cancer Gene Ther. *22*, 463–474.

39. Liu, Y.,C., Ma, W.,H., Ge, Y.,L., Xue, M.,L., Zhang, Z., Zhang, J.,Y., Hou, L., and Mu, R.,H. (2016). RNAi-mediated gene silencing of vascular endothelial growth factor C suppresses growth and induces apoptosis in mouse breast cancer *in vitro* and *in vivo*. Oncol. Lett. *12*, 3896–3904.

40. Quanz, M., Berthault, N., Roulin, C., Roy, M., Herbette, A., Agrario, C., Alberti, C., Josserand, V., Coll, J.,L., et al. (2009). Small-molecule drugs mimicking DNA damage: a new strategy for sensitizing tumors to radiotherapy. Clin. Cancer Res. *15*, 1308–1316.

41. Liu, J., Zhang, Y., Zeng, Q., Zeng, H., Liu, X., Wu, P., Xie, H., He, L., Long, Z., Lu, X., et al. (2019). Delivery of RIPK4 small interfering RNA for bladder cancer therapy using natural halloysite nanotubes. Sci. Adv. *5*, eaaw6499.

42. Tang, T., Deng, Y., Chen, J., Zhao, Y., Yue, R., Choy, K.,W., Wang, C.,C., Du, Q., Xu, Y., Han, L., and Chung, T.,K. (2016). Local administration of siRNA through microneedle: optimization, bio-distribution, tumor suppression and toxicity. Sci. Rep. *6*, 30430–30438.

43. Oh, K., Lee, O.,Y., Shon, S.,Y., Nam, O., Ryu, P.,M., Seo, M.,W., and Lee, D.,S. (2013). A mutual activation loop between breast cancer cells and myeloid-derived suppressor cells facilitates spontaneous metastasis through IL-6 trans-signaling in a murine model. Breast Cancer Res. *15*, R79.

44. Zerdes, I., Wallerius, M., Sifakis, E.,G., Wallmann, T., Betts, S., Bartish, M., Tsesmetzis, N., Tobin, N.,P., Coucoravas, C., et al. (2019). STAT3 activity promotes programmed-death ligand 1 expression and suppresses immune responses in breast cancer. Cancers *11*, 1479.

45. Su, Y.,L., Banerjee, S., White, S.,V., and Kortylewski, M. (2018). STAT3 in tumor-associated myeloid cells: multitasking to disrupt immunity. Int. J. Mol. Sci. *19*, 1803.

46. Avalle, L., Raggi, L., Monteleone, E., Savino, A., Viavattene, D., Statello, L., Camperi, A., Stabile, S.,A., Salemme, V., De Marzo, N., et al. (2022). STAT3 induces breast cancer growth via ANGPTL4, MMP13 and STC1 secretion by cancer associated fibroblasts. Oncogene *41*, 1456–1467.

47. Kemme, C.,A., Nguyen, D., Chattopadhyay, A., and Iwahara, J. (2016). Regulation of transcription factors via natural decoys in genomic DNA. Transcription *7*, 115–120.

48. Egusquiaguirre, S.P., Yeh, J.E., Walker, S.R., Liu, S., and Frank, D.A. (2018). The STAT3 target gene TNFRSF1A modulates the NF-κB pathway in breast cancer cells. Neoplasia *20*, 489–498.

49. Hammond, S.M., Aartsma-Rus, A., Alves, S., Borgos, S.E., Buijsen, R.A.M., Collin, R.W.J., Covello, G., Denti, M.A., Desviat, L.R., Echevarría, L., et al. (2021). Delivery of oligonucleotide-based therapeutics: challenges and opportunities. EMBO Mol. Med. *13*, e13243.

50. Kortylewski, M., and Moreira, D. (2017). Myeloid cells as a target for oligonucleotide therapeutics: turning obstacles into opportunities. Cancer Immunol. Immunother. *66*, 979–988.

OMTN, Volume *29*

Supplemental information

DNA minicircles as novel STAT3 decoy oligodeoxynucleotides endowed with anticancer activity in triple-negative breast cancer

Geoffrey Casas, Federico Perche, Patrick Midoux, Chantal Pichon, and Jean-Marc Malinge

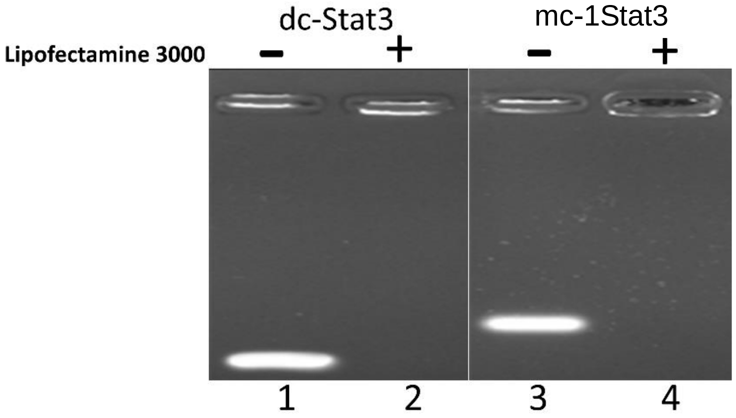

Figure S1. Gel retardation electrophoresis of linear (dc-Stat3) and MC (mc-1Stat3) DNA decoy confirms the condensation of these nucleic acids species with lipofectamine 3000. In the presence of this cationic liposome (lanes 2, 4), no more detectable band of free nucleic acid was observed while unformulated nucleic acids migrated freely as shown in lanes 1 and 3.

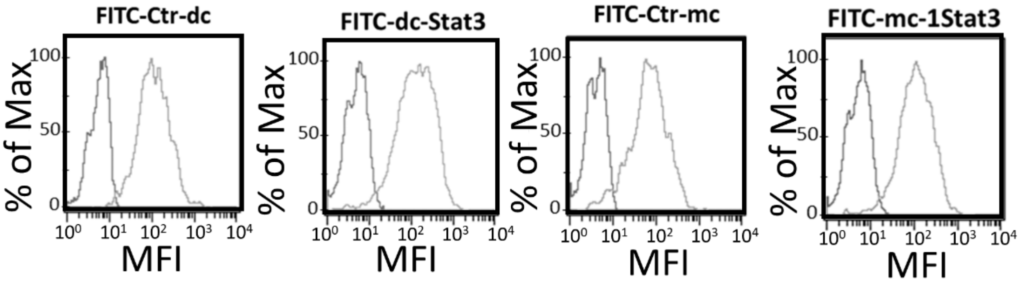

Figure S2. Cellular uptake is the same for formulated linear and DNA ODN. TNBC cancer cells were transfected in the presence of 0 nM (dark line) and 15 nM (light line) of various fluorescein-labeled linear and MCs ODNs as indicated on the panels. Flow cytometric analysis of cellular uptake were performed 6 hours after beginning treatment with lipofectamine 3000 formulated ODNs. These results show that the different specific ODNs used herein were equally taken up by the cells as their respective control ODN counterparts.

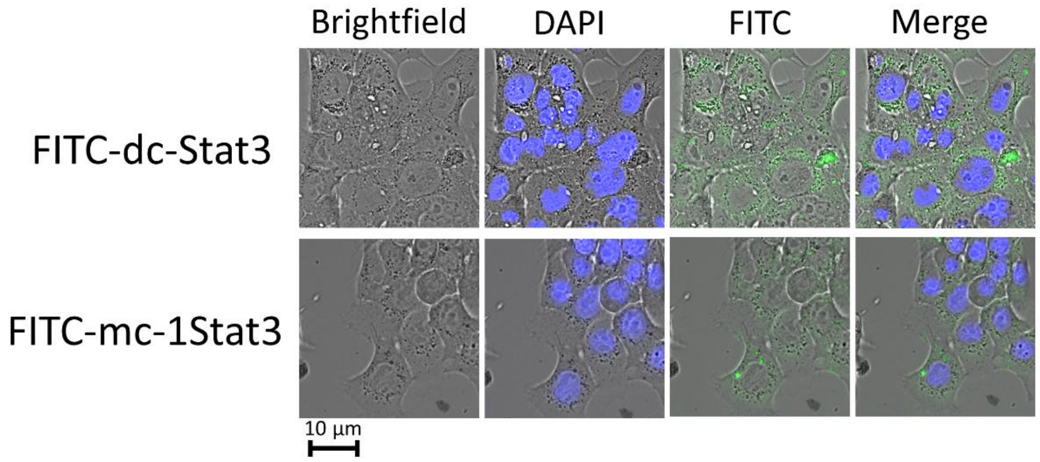

Figure S3. Comparative uptake of fluorescein-labeled dc-Stat3 and mc-1Stat3 by 4T1. 6 hours post-transfection with lipofectamine 3000 formulated ODN complexes (15 nM), confocal imaging was used with or without DAPI staining (blue) to compare intracellular delivery of both FITC-labeled linear decoy dc-Stat3 and minicircle mc-1Stat3 (green).

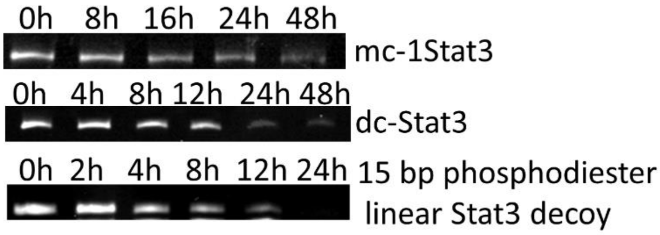

Figure S4. Biostability of 95 bp MC versus 15 bp double-stranded linear ODN in cell extract: image of denaturating gel electrophoresis showing time dependent stability of mc-1Stat3, dc-Stat3 and 15 bp phosphodiester linear STAT3 decoy in cellular extract. After incubation in cellular extract at 37°C, aliquots were taken as a function of time and loaded on denaturating polyacrylamide gel. Next, the bands were quantified following gel staining.

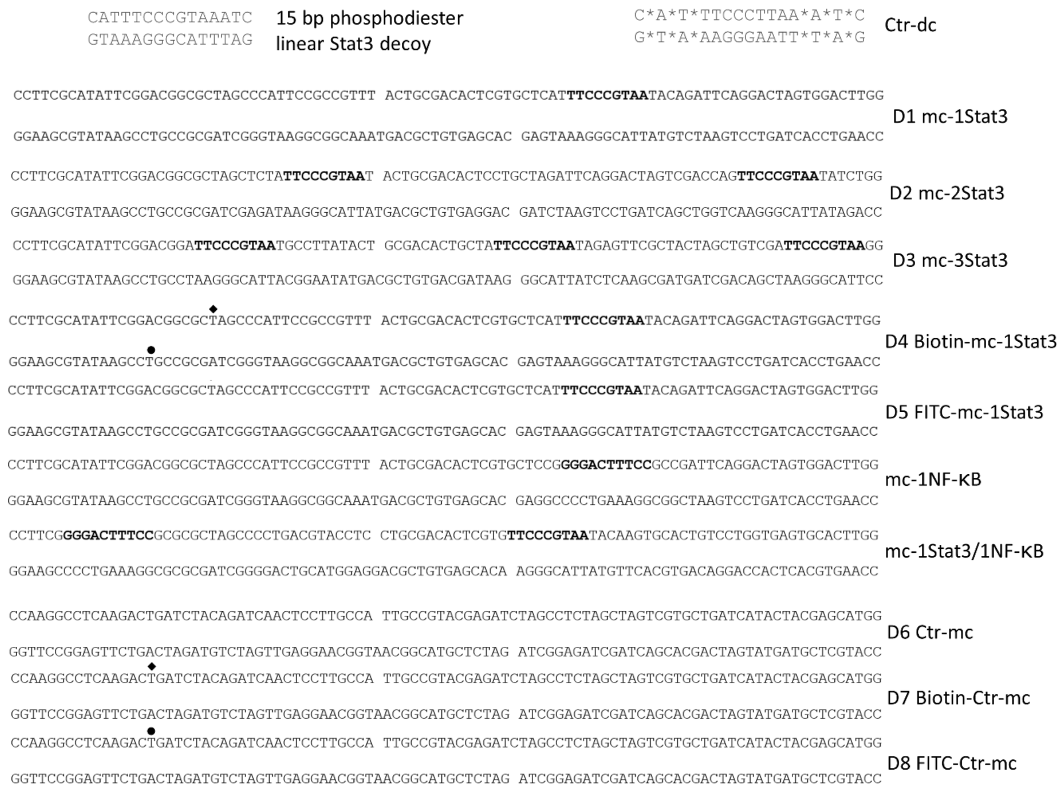

Figure S5. Sequence of DNA duplexes used in this study. 15 bp phosphodiester linear STAT3 decoy and Ctr-dc ODNs were used without further modification. Asteriks indicate phosphorothioate linkage present in linear Ctr-dc. Blunt-ended overlapping nicked 95 bp DNA duplexes oligonucleotide (D1 to D8) have been used for both the preparation of various minicircles (MCs) and two linear 95 pb DNA duplexes according to separate experimental procedure (see Material and Methods). D1 : mc1-Stat3 or 95 bp linear Stat3 DNA decoy; D2: mc-2Stat3; D3 :mc-3Stat3; D4: biotinylated mc-1Stat3; D5 : FITC-mc-1Stat3; D6: Ctr-mc or linear 95 bp; D7: biotinylated Ctr-mc; D8: FITC-Ctr-mc. Nick is represented by an interruption within nucleotide sequence in either top or bottom strand giving rise to an overlapped double-stranded region allowing higher DNA bendability and hence efficient circularization reaction for minicircle production. With duplexes D1 to D5, the nucleotides shown in bold indicate the presence of one (D1, D4), two (D2) and three (D3) customized STAT3 binding sequence. With the duplexes D4 and D7, (◆) denotes the presence of a biotin residue attached to position 5 of thymine; with the duplexes D5 and D8, (●) indicates the presence of a fluorescein residue (FITC) attached to position 5 of thymine ring by a 6-carbons spacer arm.

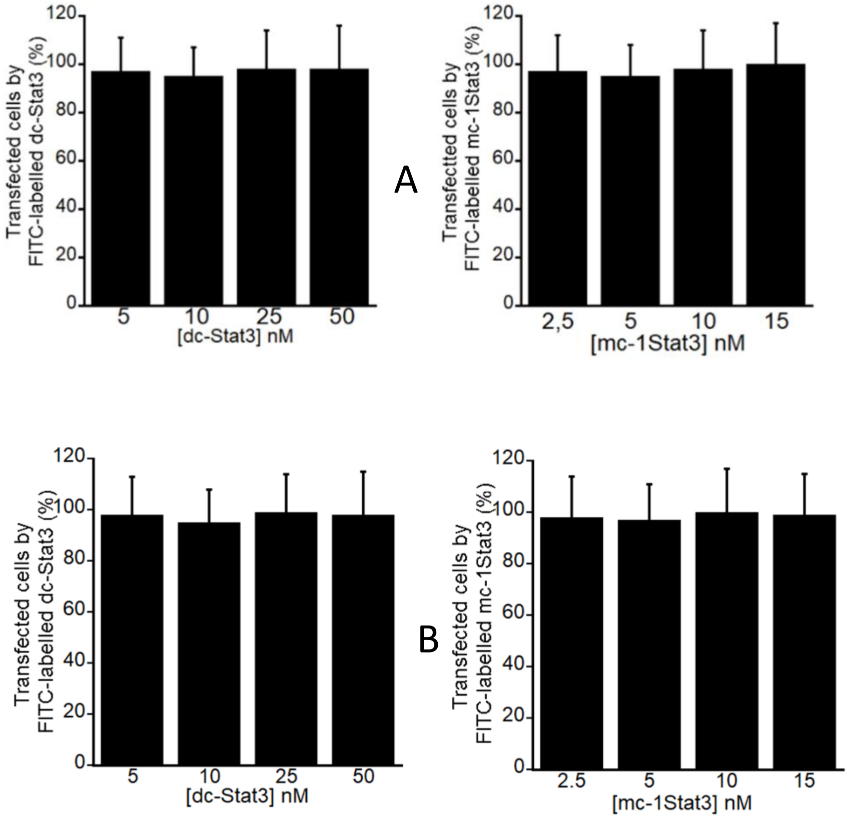

Figure S6. Cell transfection efficiency is independent of STAT3 linear and MC decoy concentration. MDA-MB-231 (A) or 4T1 (B) cells were transfected in the presence of increasing concentration of fluorescent-labelled ODN formulated with lipofectamine 3000 and 24 hours later the percentage of positive fluorescent cells was determined by flow cytometry.

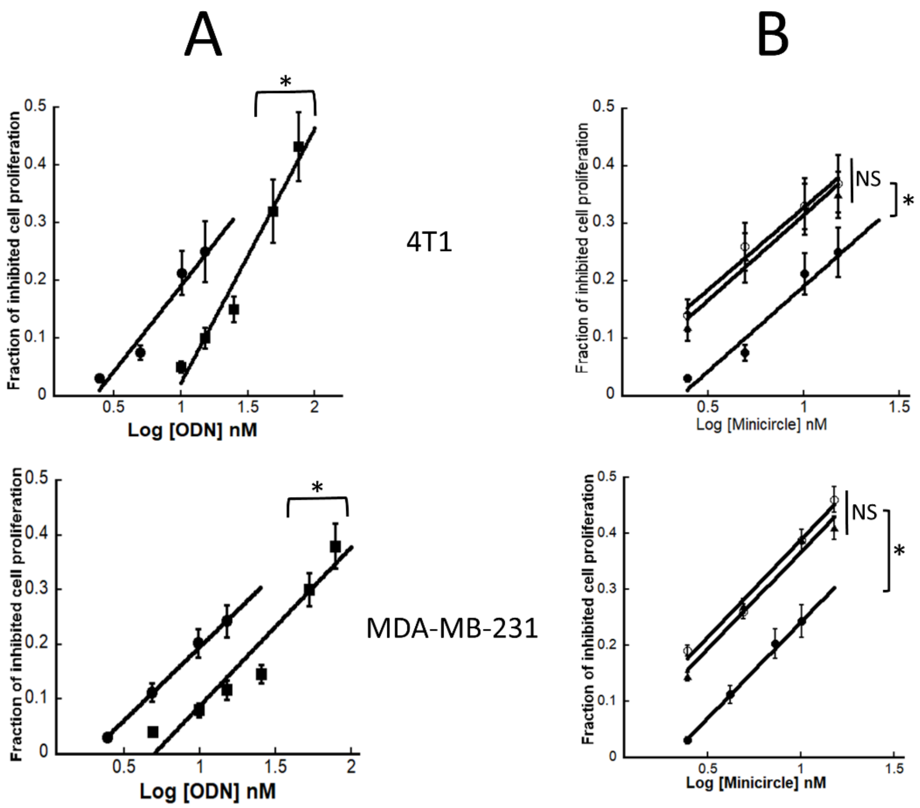

Figure S7. Linearization of cell proliferation dose-response curve. (A) Comparison of TNBC cells treatment with dc-Stat3 versus mc-1Stat3; (B) TNBC cells treatment with MC containing one, two or three STAT3 binding sites. (■) dc-Stat3; (●) mc-1Stat3 (▲) mc-2Stat3; (○) mc-3Stat3. Linearization was performed by plotting the fraction of inhibited cell proliferation as a function of logarithm base 10 of nucleic concentration (1). The concentration of each nucleic acids that induced 20% of inhibition (IC20) was then calculated from the corresponding dose response curve. All the data are expressed as the means ±SD (**P* < 0.05 ; ** *P* < 0.01; *** *P* < 0.001; NS, not significant).

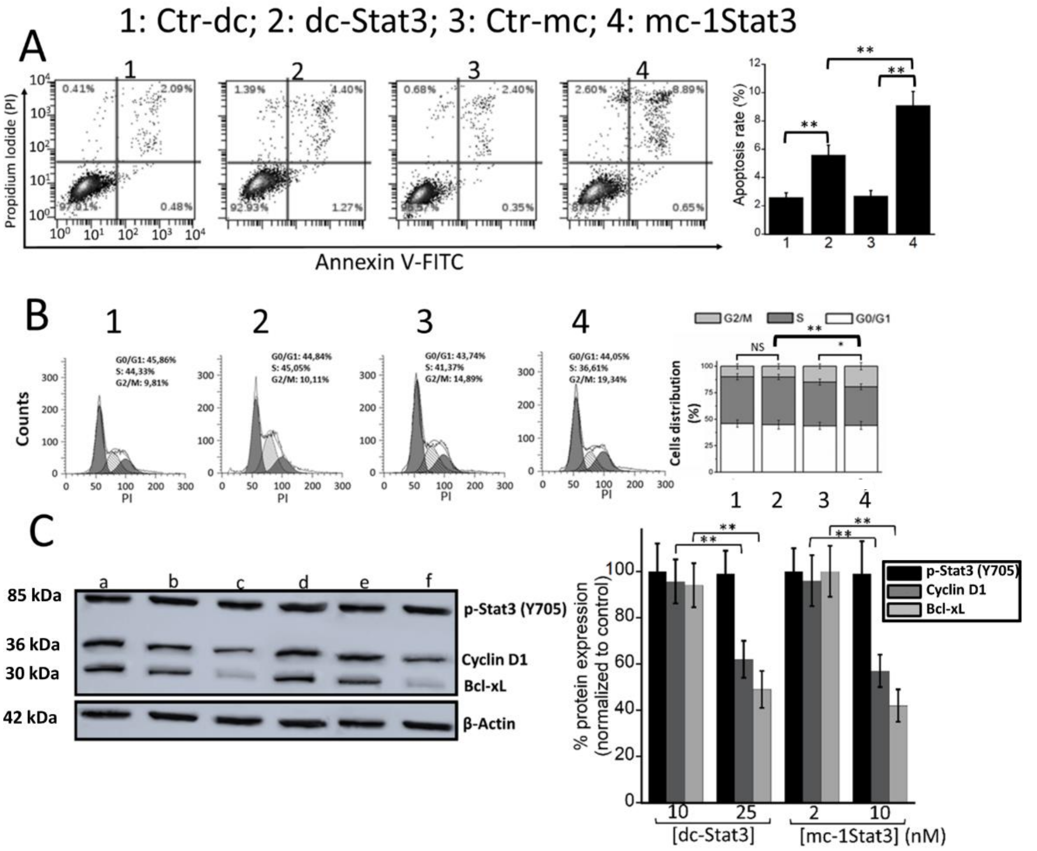

Figure S8. mc-1Stat3 triggers apoptosis, induces G2/M phase cell cycle arrest and inhibits Stat3-dependent gene expression more efficiently than dc-Stat3 in 4T1 cells: (A) Detection of apoptotic 4T1 cells by flow cytometry analysis using Annexin V-FITC and propidium iodide: cells were transfected with 15 nM of mc-1Stat3, dc-Stat3, Ctr-mc or Ctr-dc and then stained with annexin V-FITC and propidium iodide 48 h later. Result of apoptosis assays is shown on the right side as histogram, 4T1 cells positive for both Annexin V and PI being considered as apoptotic cells. (B) 4T1 cell cycle progression analysis: 4T1 cells were transfected as in (A) and cell cycle distribution was then assessed using flow cytometry. Representative results of the cell cycle analyses and histogram of cell cycle distribution are shown on the left and on the right, respectively. (C) Expression level of p-STAT3 (Y705), Bcl-xL, Cyclin-D1 following 4T1 cells treatment with mc-1Stat3 and dc-Stat3. 4T1 were treated with dc-Stat3 or mc-1Stat3 and total cell extract was obtained 48 h later after cell lysis. Protein expression was assessed by western blot as indicated on the left side of Figure C. Lane (a): Ctr-dc; lane (b) and (c): dc-Stat3, 10 and 25 nM, respectively; lane (d): Ctr-mc; lane (e) and (f): mc-1Stat3, 2 and 10 nM, respectively. β-actin protein was used as internal loading control. On the right side of Figure C is shown the quantified expression levels of proteins as a function of the nature and dose of decoy ODNs as indicated. Results presented are representative of three independent experiments performed in triplicates. All the data are expressed as the means ±SD of three independent experiments (*$P < 0.05$; ** $P < 0.01$; *** $P < 0.001$; NS, not significant).

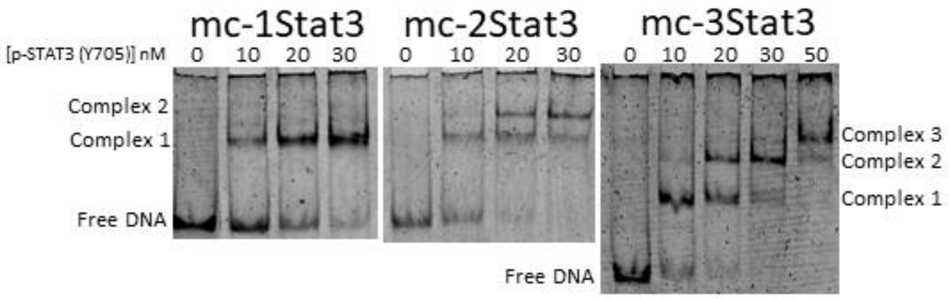

Figure S9. Titration of STAT3 by decoy MC increases as a function of the number of DNA binding sites. Each type of minicircle was incubated with increasing concentrations of p-STAT3 (Y705) protein as indicated and the reaction mixture analyzed by electrophoretic mobility shift assay (EMSA). Free DNA indicates migration of MC in the absence of protein. The shifted band named complex 1, complex 2 and complex 3 corresponds to gel-shifted complex containing one, two and three proteins per minicircle, respectively. Near complete saturation of the binding sites by STAT3 is reached for the three MCs as shown by the presence of a major shifted band. The binding assay and the DNA binding activity of p-STAT3 (Y705) was performed by EMSA as described (2). Recombinant histidine-tagged p-STAT3 (Y705) protein was produced using plasmid pet28a STAT3 βTC vsv his tag (a kind gift from Prof. Thorsten Berg, University of Leipzig, Germany) and purified as previously described (3).

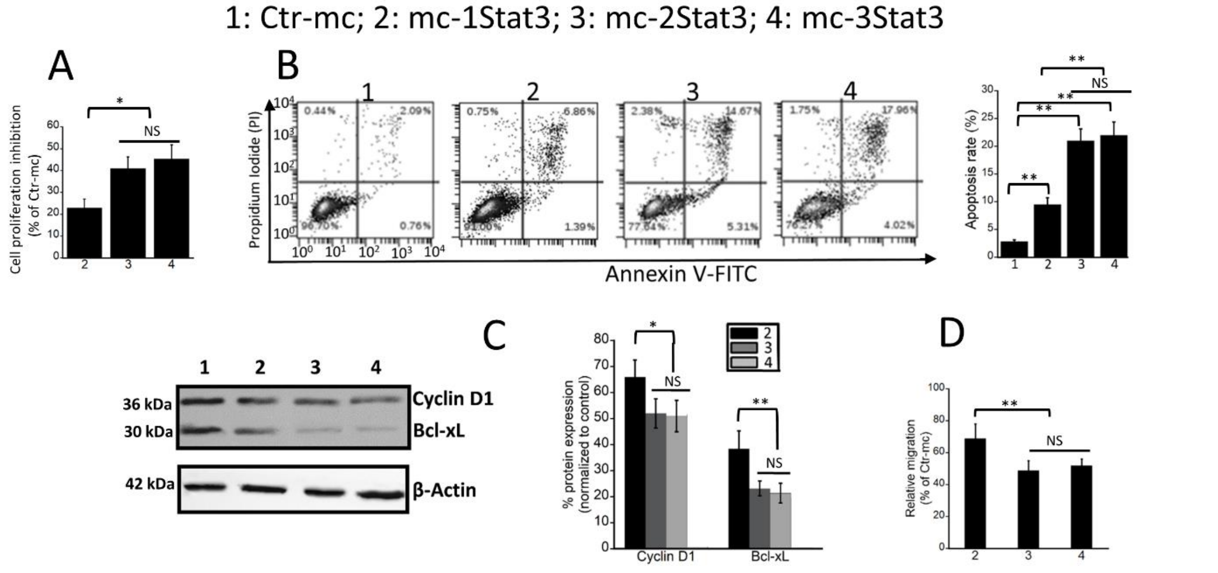

Figure S10. Effect of minicircle STAT3 binding sites number on 4T1 cellular activity.

A) Comparative proliferation evaluation of 4T1 cells treated with mc-1Stat3, mc-2Stat3, mc-3Stat3 (10 nM); cell survival was determined 48 h after using Alamar Blue assay. B) Detection of apoptotic 4T1 cells by flow cytometry analysis using Annexin V-FITC and propidium iodide. 4T1 cells were treated with 10 nM of mc-1Stat3, mc-2Stat3, mc-3Stat3 and Ctr-mc; 48 h later, the level of apoptosis was evaluated using the AnnexinV/PI dual-labeling technique, as determined by flow cytometry. Quantitative results of apoptosis assays is shown on the right side as histogram. C) Expression level of Bcl-xL and Cyclin-D1 following 4T1 cells treatment with mc-1Stat3, mc-2Stat3 and mc-3Stat3**.** Western blot assay of 4T1 treated with minicircles bearing one to three STAT3 binding sites and with Ctr-mc at 10 nM concentration. Cell lysates were immunoblotted for the indicated proteins. β-actin was used as a loading control. D) Migration inhibition of 4T1 cells induced by STAT3 MCs. 4T1 cells were first treated with 10 nM concentration of minicircle and 24 hours post-treatment, migration was determined using Transwell migration assay. After 6 hours, the migratory cells were counted and the quantification is shown on histogram analysis. Results presented are representative of three independent experiments performed in triplicates. All the data are expressed as the means ±SD of three independent experiments, (**P* < 0.05 ; ** *P* < 0.01; *** *P* < 0.001; NS, not significant).

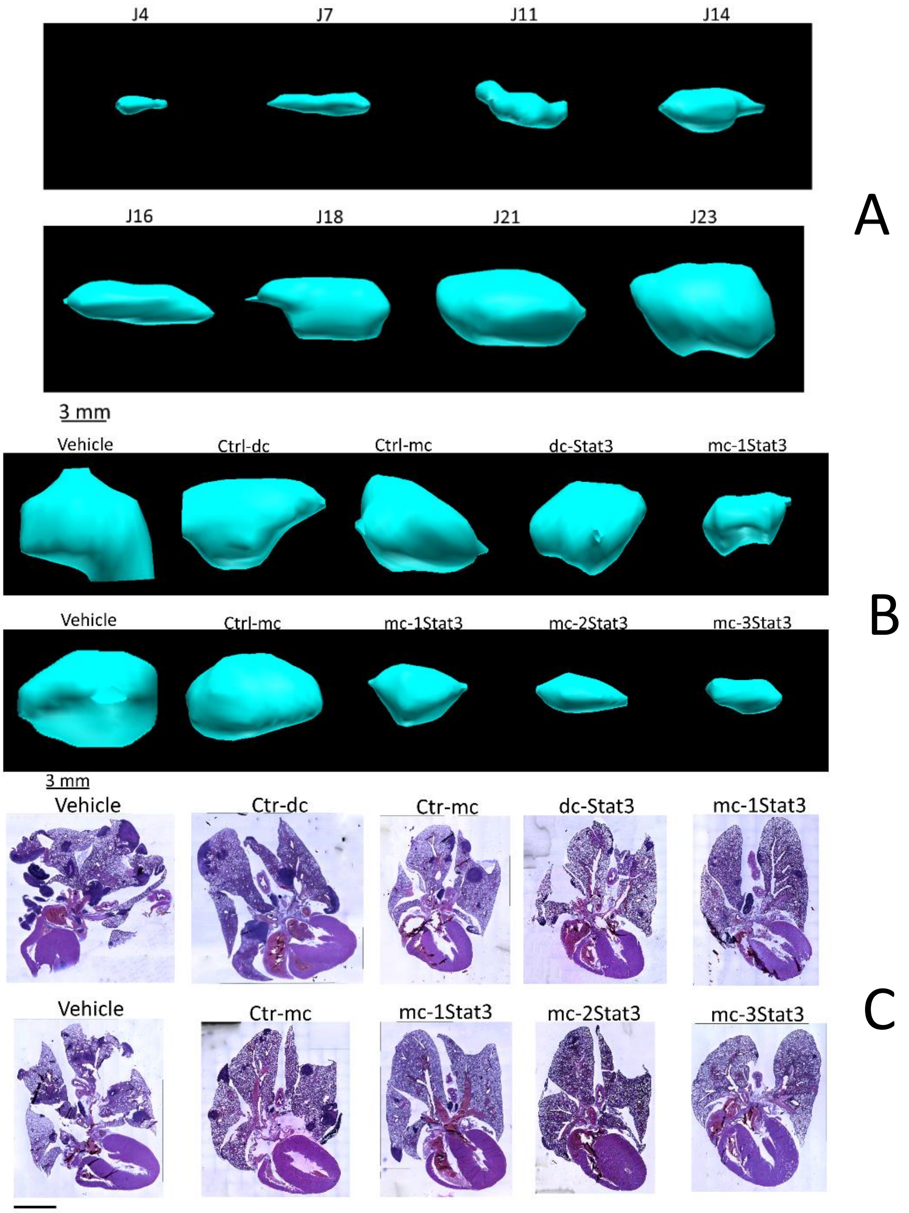

Figure S11. Representation of primary 4T1 tumor 3D reconstruction of ultrasound imaging data and H&E staining of lung section illustrating *in vivo* data obtained with or without treatment. Examples showing A) the time-dependent change in tumor size for one mice belonging to the vehicle group, B) the mice group-dependent primary tumor size as a function of treatment at 26 days following 4T1 cells inoculation within mammary fat pad, C) the mice group-dependent lungs sections used to visualize metastatic foci (dark purple) as a function of treatment (Scale bar = 1 cm). Vevo Labs and ImageJ softwares were used for production of 3D images following tumor volume measurement and surface area measurement of metastatic foci, respectively.

Reference:

1) Chou, T., C. (2006). Theoretical basis experimental design and computerized simulation of synergism and antagonism in drug combination studies. Pharmacological reviews, *58*, 621-681.
2) Thibault, T., Degrouard, J., Baril, P., Pichon, C., Midoux, P., Malinge, J.-M., (2017). Production of DNA minicircles less than 250 base pairs through a novel concentrated DNA circularization assay enabling minicircle design with NF-κB inhibition activity. Nucleic Acids Res. *45*, e26.
3) Becker, S., Corthals, G.,L., Aebersold, R., Groner, B., Müller, C., W., (1988). Expression of a tyrosine phosphorylated, DNA binding Stat3beta dimer in bacteria. FEBS Lett. *441*, 141-147.
